# Comparative analysis of the response and gene regulation in cold resistant and susceptible tea plants

**DOI:** 10.1371/journal.pone.0188514

**Published:** 2017-12-06

**Authors:** Qiuyan Ban, Xuewen Wang, Cheng Pan, Yiwei Wang, Lei Kong, Huiguang Jiang, Yiqun Xu, Wenzhi Wang, Yuting Pan, Yeyun Li, Changjun Jiang

**Affiliations:** 1 State Key Laboratory of Tea Plant Biology and Utilization/key Laboratory of Tea Biology and Processing, Ministry of Agriculture, Anhui Agricultural University, Hefei City, Anhui Province, PR China; 2 Department of Genetics, University of Georgia, Athens, Georgia, United States of America; 3 Henan Provincial Key Laboratory of Tea Plant Biology, Xinyang Normal University, Xinyang, Henan Province, PR China; Universite Paris-Sud, FRANCE

## Abstract

Cold environment is the main constraint for tea plants (*Camellia sinensis*) distribution and tea farming. We identified two tea cultivars, called var. *sinensis* cv. *Shuchazao* (SCZ) with a high cold-tolerance and var. *assamica* cv. *Yinghong9* (YH9) with low cold-tolerance. To better understand the response mechanism of tea plants under cold stress for improving breeding, we compared physiological and biochemical responses, and associated genes expression in response to 7-day and 14-day cold acclimation, followed by 7-day de-acclimation in these two tea cultivars. We found that the low EL50, low Fv/Fm, and high sucrose and raffinose accumulation are responsible for higher cold tolerance in SCZ comparing with YH9. We then measured the expression of 14 key homologous genes, known as involved in these responses in other plants, for each stages of treatment in both cultivars using RT-qPCR. Our results suggested that the increased expression of *CsCBF1* and *CsDHNs* coupling with the accumulation of sucrose play key roles in conferring higher cold resistance in SCZ. Our findings have revealed key genes regulation responsible for cold resistance, which help to understand the cold-resistant mechanisms and guide breeding in tea plants.

## Introduction

Low temperature is a major constraint on the growth, geographical distribution, and yield of some plants. Cold resistance of many plants[1–4], e.g. *Eucalyptus nitens*, *Miscanthus*, *Medicago sativa* and *North American Rhododendron* can be improved by prior exposure to a period of low, nonfreezing temperatures, which known as cold acclimation (CA) [5–7]. For instance, CA improves the tolerance of North American Rhododendron from -7°C to -53°C [4]. Up to date, CA is a key strategy to increase the physiological adaptation of tea plants to low temperatures [8]. During CA, many physiological and biochemical processes are altered in plants. Those processes include the cytoskeleton rearrangement as an integrating system perceiving the signals [9], accumulated membrane phospholipids and modifications in lipid composition of different organelles. For example, the proportion of MGDG (monogalactosyldiacylglycerols) was decreased and the proportion of DGDG (digalactosyldiacylglycerols) was increased in the chloroplast in Rye [10–14]. Moreover, plants introduce the accumulation of antifreeze proteins and cryoprotectants like soluble sugars and proline [15–16]. The increased synthesis of soluble sugars, including sucrose, glucose, raffinose, and fructose, contributes directly to membrane stabilization in *Alcantarea imperiali* [17], and *Camellia sinensis* [8]. The raised content of proline in *Triticum aestivum* [18], *Arabidopsis* [19] and *Camellia sinensis* [8] was also observed during CA. Antioxidant metabolism is known to improve the scavenging activity of reactive oxygen species (ROS) and maintain redox balance during CA [20]. During CA, a high ratio of abscisic acid (ABA) to gibberellin content has been shown to increase freezing tolerance in some woody taxa [21].

Upon cold stress, the expression of various cold-regulated (COR) genes are induced to protect plants [22]. The expression of COR genes is regulated by both the CBF (C-repeat-binding factor)-mediated ABA-independent pathway and the bZIP (basic region/leucine zipper)-mediated ABA-dependent pathway [23]. CBF transcription factors regulate ~12% of the cold-responsive transcriptome [24]. ICE1 (inducer of CBF expression 1)-CBF-COR cold-response pathway in plants is critical for configuring cold-induced transcriptomic changes [25–26]. Genes of the ICE1-CBF cold-response pathway have been reported in woody and herbaceous plants [27–29]. Studies have shown that the cascade regulation of *ICE1*, *CBF*, and *COR* is the main pathway for cold acclimation [30–31]. In *Arabidopsis*, *ICE1* express constitutively and is not responsive to cold stress, whereas ICE1 undergoes sumoylation to become functionally active [32]. Three CBFs (*CBF1-3*) were found in *Arabidopsis*. *CBF1* and *-CBF3* positively regulates the downstream CBF-target genes, while *CBF2* negatively regulates them [33]. Wang at al. [34] found that the ICE1-CBF-COR pathway was conserved in tea plants. To date, several COR genes have been discovered in tea plant including one *CsICE1* (FE861156), two *CsCBFs*, designated as *CsCBF1* (EU563238), *CsCBF2* (KC702795), and three dehydrin homologs designated as *CsDHN1* (GQ228834.1), *CsDHN2* (FJ436978) and *CsDHN3* (KY270880) [34–36].

Several studies revealed the key enzymes’ activities during sugar synthesis, and associated genes expression during CA in plants. Sucrose is synthesized in the cytosol by the sucrose-phosphate synthase (SPS) and degraded by either sucrose synthase or invertase (INV) into a monosaccharide or derivative [37]. Raffinose synthase (RS) for raffinose synthesis was also explored in recent researches upon cold stress [38]. Yue et al. [8] analyzed the expression patterns of 32 genes during the natural CA in tea plant (var. *sinensis* cv. *Longjing43*) and found that expression of *CsSPS*, *CsINV5 and CsRS2* was significantly induced. To date, it is known that the proline biosynthesis is catalyzed by P5C synthase (P5CS) and P5C reductase (P5CR) in plants [39–40]. Another key enzyme in the proline synthesis pathway is Ornithine-D-aminotransferase (OAT) [41]. Degradation of proline is catalyzed by Pro-dehydrogenase (ProDH) and P5C-dehydrogenase (P5CD) [42]. In tea plants, the sequences of *CsP5CS* (KJ143742.1), *CsOAT* (KJ641844.1) and *CsP5CR* (KY368574), *CsP5CDH*(KY368572) and *CsProDH* (KY368573) are available at NCBI (https://www.ncbi.nlm.nih.gov/).

Tea plants (*Camellia sinensis* (L.) O. Kuntze), one of the important economic wooden plants in the world, are mainly grown in subtropical and tropical regions. Two basic classes of varieties can be classified as var. *assamica*, a quick-growing tree well suited to tropical climates, and var. *sinensis*, a slower-growing bush that can withstand colder climates than *assamica* [43–44]. Tea plants are vulnerable to cold injury during winter such as in East Asia (China, Japan), especially in northern China. Recent studies have explored the response of tea plants to cold stress and natural CA [45–48]. However, a comparative study on cold resistance between cold-resistant and cold-susceptible cultivars has not been reported yet. The present study was conducted to explore the molecular mechanism of cold resistance by treating the cold-resistant *camellia var*. *sinensis* CV. *Shuchazao* (SCZ) and cold-susceptible *camellia var*. *assamica* CV *Yinghong9* (YH9) under CA and de-acclimation (DA). We found difference in biochemical changes, including EL50 (temperature leading to 50% tissue damages due to leakage of electrolyte), Fv/Fm (maximum quantum yield of PSII photosystems), sugars and proline. Then we examined the expression of 14 genes related to these biochemical changes. Comparison of gene expression and study of biochemical changes in the responses to cold in two tea cultivars led to our finding of the difference in cold tolerance. Our results indicated that the increased expression of *CsCBF1* and *CsDHNs* coupling with the accumulation of sucrose has played a role in conferring higher cold resistance in tea cultivar SCZ. The results provide understanding in biochemical and gene regulatory mechanisms of cold resistance in tea plants.

## Materials and methods

### Plant material

The clone cuttings of *Camellia sinensis* cv. *Shuchazao* and *Camellia sinensis* var. *assamica* cv.*Yinghong9* were obtained from the Dechang Tea Plantation in Anhui (116° 56' 24'' E, 31° 27' N) and the Tea Research Institute of Guangdong Academy of Agricultural Sciences (113° 22' 48'' E, 24° 10' 12'' N), China, respectively. One-year-old cutting-propagated plants were transferred to a growth chamber with temperature cycles of 25°C at day time and 20°C at night time, 12 h photoperiod, and 70% relative humidity for one month. Subsequently, they were subjected to varying degrees of cold acclimation and de-acclimation. Ten well-grown one-year-old tea plants were collected and used as non-acclimation (NA). The following cold acclimation (CA) treatments were applied in this study: CA1 was conducted by exposing SCZ and YH9 to low temperature (10/4°C, day/night temperature) for 7 days. Afterwards, CA2 was conducted by exposing SCZ and YH9 to lower temperatures (4/0°C, day/night temperature) for another 7 days. Lastly, the plants were exposed to normal temperature (25/20°C, day/night temperature) for 7 days for de-acclimation. At each time point, the leaves were collected, immediately frozen in liquid nitrogen and stored at −80°C until use. Three biological replicates were conducted.

### Electrolyte leakage assay

Relative electrolyte leakage was measured to evaluate the cell membrane damage as described with some modifications [49]. Briefly, after washing with distilled deionized water, the leaf pieces were obtained using a puncher from leaves after each treatment. After subsequently exposed to -2°C, -4°C, -6°C, -8°C, and -10°C for 12 hours, samples were placed in glass bottles containing 20 mL of distilled deionized water. The electrical conductivity of the solution (L1) was determined using a conductivity meter STARTER 3100C (Ohaus; America) at 25°C. The solutions were then heated to 100°C for 30 min and the final electrical conductivity (L2) was determined after cooling to 25°C. The REC (relative electrical conductivity) was calculated as L1/L2×100%.

### Fv/Fm

Mature leaves (from third to fifth leaves) of tea cultivar SCZ and YH9 were carefully clamped in the middle part of the leaves, avoiding the main vein and then dark-adapted in leaf clips for 20 min prior to measurement. Chlorophyll fluorescence parameters Fm and Fo were measured by OS-30P modulated fluorometer (Opti-Sciences, USA) and Fv was obtained using Fv = Fm-Fo [50]. Ten biological replicates were performed for the experiment.

### Measurement of proline content

Proline contents in SCZ and YH9 were measured by the colorimetric assay according to Bates method with some modifications [51]. Briefly, approximately 0.5 g leaves of SCZ and YH9 were ground into fine powder in liquid nitrogen. The powder was immediately resuspended in 5 mL of 4% sulfonic acid and sonicated for 30 min. The mixture was subsequently centrifuged for 30 min at 12000 rpm and the supernatant was collected. 2 mL supernatant, 2 mL glacial acetic acid and 3 mL ninhydrin reagent (2.5% [w/v] ninhydrin, 60% [v/v] glacial acetic acid, and 40% 6 M phosphoric acid) were added, mixed and heated to 100°C for 40 min. After cooling down to room temperature, 5 mL toluene was added and the absorbance was measured at 520 nm using an UV Spectrophotometer (U-5100, Hitachi).

### Measurements of soluble carbohydrates

Contents of soluble carbohydrates of fructose, sucrose, glucose, raffinose and trehalose in the leaves of tea cultivar SCZ and YH9 were measured by High Performance Liquid Chromatography (HPLC) (Agilent, America). The samples were prepared following the protocol as previously described with some modifications [52]. Briefly, approximately 0.5 g leaves of SCZ and YH9 were weighed and ground in liquid nitrogen, and 10 ml of distilled water was added immediately. After heating at 100°C for 1 h, the mixture was subsequently centrifuged for 10 min at 12000 rpm and the supernatant was collected. The aqueous phase was collected and dried on a rotary evaporator. It was then resuspended in distilled water and filtered through a 0.22 μm filter membrane prior to HPLC analysis. Standard of fructose, sucrose, glucose, raffinose and trehalose were purchased from Sangon Biotech. Co. (Shanghai, China)

### RNA extraction and real-time quantitative PCR analysis

Total RNAs were extracted from leaves of tea cultivar SCZ and YH9 with RNA prep Pure Plant kit (Tiangen, Beijing, China). The total RNAs were reverse transcribed into first-strand cDNA with PrimeScript Reagent Kit (TaKaRa, Dalian, China) and the cDNAs obtained were used as templates for PCR amplification with specific primers. Gene-specific primers (Table 1) were used for real-time quantitative RT-PCR. The glyceraldehyde-3-phosphate dehydrogenase (GAPDH) gene was used as an internal reference gene [53] and the relative expression was calculated using the 2^ΔCt^ method [54]. Each reaction contained 12.5 μL of SYBR^®^Premix Ex Taq^™^II (Tli RNaseH Plus; TaKaRa, Dalian, China), 2 μL cDNA, and 1 μL 10 μM gene-specific primers in a final volume of 25 μL. All reactions were carried out using the CFX96^™^ Real-Time System (Bio-Rad, USA) using a two-step method: 95°C for 3 min; 40 cycles of 95°C for 10 s, 62°C for 30 s.

**Table 1:** Genes and corresponding primers used for the RT-qPCR experiments.

Gene name	GenBank Accession No.	Primer	Primer sequence (5'–3')
GAPDH	GE651107	Forward	TTG GCA TCG TTG AGG GTC T
		Reverse	CAG TGG GAA CAC GGA AAG C
CsICE1	FE861156	Forward	ATG TTT TGT AGC CGC AGA C
		Reverse	GCT TTG ATT TGG TCA GGA TG
CsCBF1	EU563238	Forward	AGA AAT CGG ATG GCT TGT GT
		Reverse	TTG TCG TCT CAG TCG CAG TT
CsCBF2	KC702795	Forward	CAC AGC CTG CTC ATC ACT
		Reverse	ACC ACT GCC ACA ATC TG
CsDNH1	GQ228834.1	Forward	ACA CCG ATG AGG TGG AGG TA
		Reverse	AAT CCT CGA ACT TGG GCT CT
CsDNH2	FJ436978	Forward	ACT TAT GGC ACC GGC ACT AC
		Reverse	CTT CCT CCT CCC TCC TTG AC
CsDNH3	KY270880	Forward	TCC ACA TCG GAG GCC AAA AG
		Reverse	AAC CCT CCT TCC TTG TGC TC
CsSPS	KF696388	Forward	ACC TGG AGG CGA TTC TGG ATG
		Reverse	TTC CAA ATC CGC CAG CAC ATA
CsRS2	KP053395	Forward	CGG TTT GGC GCT TAC TCT TC
		Reverse	TCT CCT CTT CTG CAA CCG GA
CsINV5	KP053402	Forward	AGT CTT GCC CCT TGA TGT CG
		Reverse	AAC CAA ACG GTC CAA GAG CA
CsP5CS	KJ143742.1	Forward	AGG CTC ATT GGA CTT GTG ACT
		Reverse	CAT CAG CAT GAC CCA GAA CAG
CsOAT	KJ641844.1	Forward	GCG GTT AAT CAG GGA CAT
		Reverse	ACA CCT TCG GCA CCA GTA
CsP5CR	KY368574	Forward	TAG GGG AGG CGG CAT CAG TT
		Reverse	ACC CCT CCA TCA GCC AAA GC
CsP5CDH1	KY368572	Forward	TGC TGA TGG GAA GAC GAT
		Reverse	GCC GAG CAC TTT TGA CCA CT
CsProDH	KY368573	Forward	CAA AAC CCA AAT CCA ACC G
		Reverse	TCC TCC TCA CTA CCC CCA AC

### Primer design

The primers were designed against the sequence of genes which is retrieved from Genbank using the listed accession number (Table 1). The software Primer Premier 5 (Premier Biosoft International, Palo Alto, California, USA) was used to designed specific primers (Table 1) and the primers were then synthesized by Sangon Biotech Co. (Shanghai, China). We checked the specify of the primers and which produced one peak in melting curve, indicating a single amplicom of target gene. Then we used these primers for the level of transcript (Figures A-O in S1 Text). qPCR products have been sequenced and the results evaluated using the DNAman computer software version 5.0 (Lynnon Biosoft) (Figures A-O in S2 Text).

### Statistical analysis

EL50 was calculated by logistic equations. Statistical analyses were performed using DPS and Prism5, GraphPad Software. The results were expressed as mean value ± standard error (SE). Different letters indicate significant differences to Duncan’s multiple range tests with *P* < 0.05.

## Results

### Cold acclimation induces difference freezing tolerance in tea cultivars

**Fig 1:**
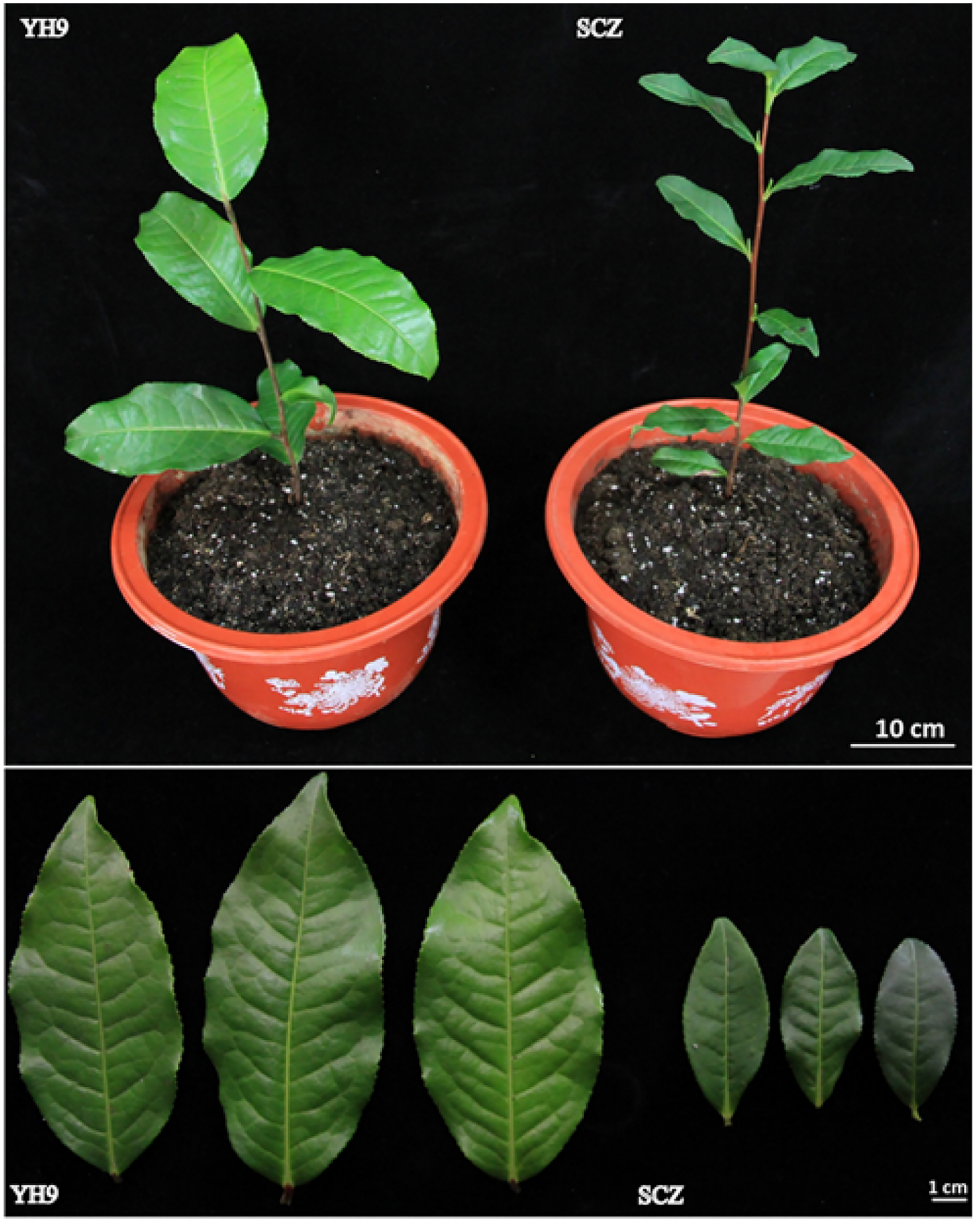
Comparison between *Camellia sinensis* cultivar YH9 and cultivar SCZ. Images were taken from one-year-old plant clonally propagated from cuttings.

**Fig 2:**
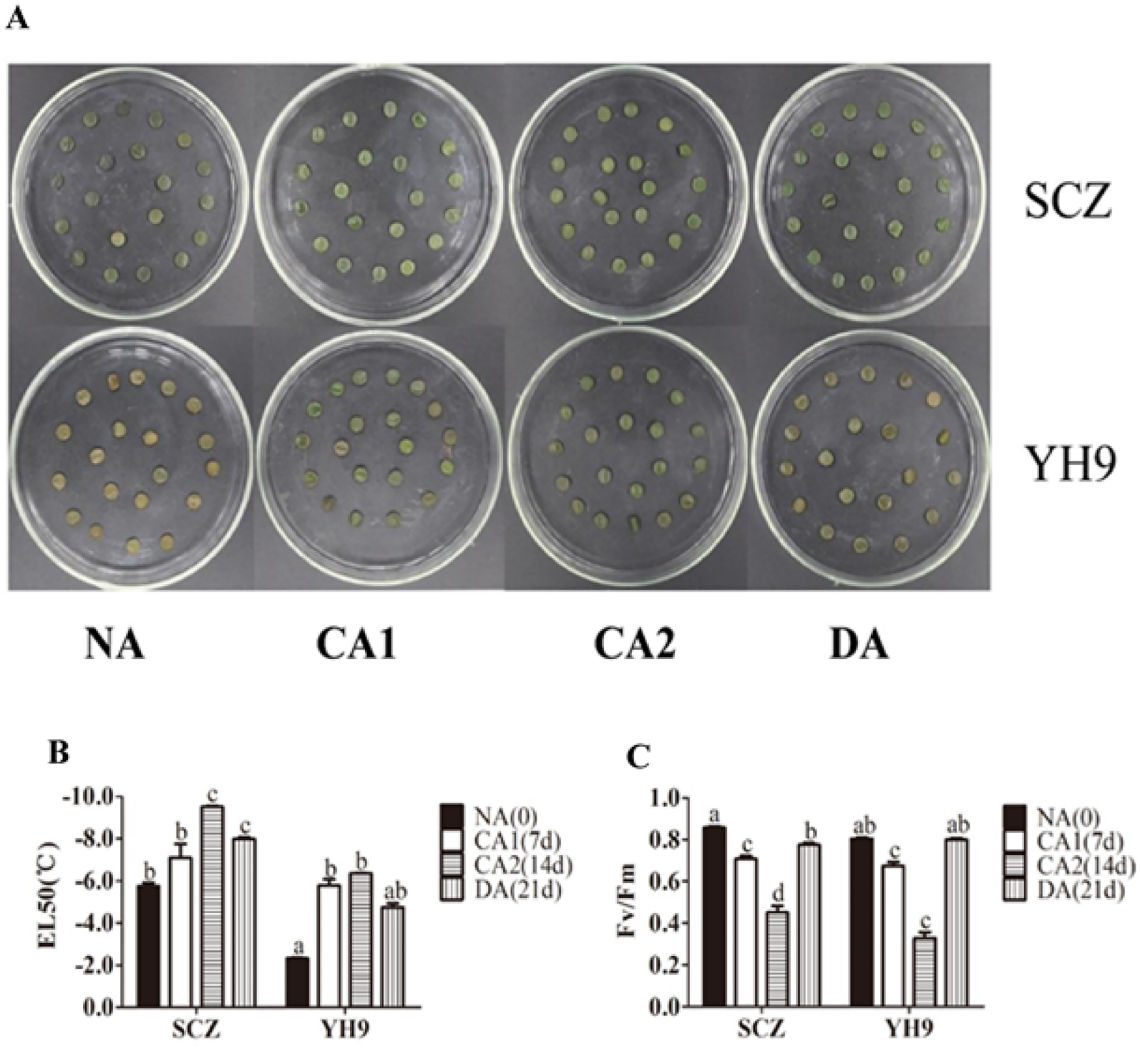
Effects of CA and DA on freezing tolerance of SCZ and YH9. (A), The detached leaf discs of SCZ and YH9 exposed to -6°C for 12 h at different stages (NA, CA1, CA2, and DA). The values EL50 (B) and Fv/Fm (C) in SCZ and YH9 changed in response to CA and DA periods. Data were displayed as the mean of three replicates with standard error. Columns with different letters in (B) or (C) had significant differences according to Duncan’s multiple range tests with *P* < 0.05. NA: non-acclimation; CA1: cold acclimation of 7 days at 10/4°C, day/night temperature; CA2: cold acclimation of 7 days at 4/0°C, day/night temperature; DA: de-acclimation of 7 days at 25/20°C, day/night temperature.

To investigate the cold tolerance, we selected two tea cultivars SCZ and YH9 which are known as with high and low cold tolerance, respectively (Fig 1). SCZ has been planted in cool areas in middle and warm areas in south China while cultivar YH9 in warm areas only in south China. Tea cultivar SCZ has smaller leaf than cultivar YH9 (Fig 1). We treated the one-year-old plants clonally propagated from cuttings of these two tea cultivars SCZ and YH9 with cold treatments CA1, CA2 and DA in growth camber to measure the physiological responses, membrane damage and chlorophyll content. We first treated the tea cultivar SCZ under 10/4°C (day/night) for 28 days and checked the change Fv/Fm and total sugar contents in a time course manner. We found that 7 days of cold treatment is enough to detect significant changes (S3 Text). Therefore, we used 7 days of treatment in this study. Our results showed that SCZ leaves remain green while those of YH9 became reddish brown after all treatments (Fig 2A). We further examined the electrolyte leakage which reflects cell membrane damage in cold by using EL50 analysis. As shown in Fig 2B, the EL50 had significant difference between SCZ and YH9, and the EL50 were -5.7°C and -2.3°C in SCZ and YH9 before cold treatment, respectively. Cold treatment CA1 treatment resulted in reduced EL50 values for both SCZ and YH9 (Fig 2B). Further cold treatment CA2 led to a more reduction of EL50 in SCZ to -9.4°C, while the EL50 value of YH9 remained unchanged compared with CA1. Lower EL50 represents less leakage. Thus, this result suggested higher cold tolerance in cultivar SCZ. The Fv/Fm value of both cultivars was relatively consistent in the range of 0.80~0.85 before treatment while both SCZ and YH9 displayed similar lower Fv/Fm values (*P* < 0.05) after CA1 treatment (Fig 2C). CA2 treatment further reduced the Fv/Fm value but the value of YH9 reduced more than SCZ (*P* < 0.05) (Fig 2C). The lower Fv/Fm suggests less chlorophyll, which explains the observed reddish color in YH9 after treatment. After DA treatment, the ratios of Fv/Fm in the two cultivars returned to the normal level (Fig 2C), which supported the Fv/Fm change in chlorophyll was caused by cold treatment and was reversible.

### Effect of CA and DA on soluble sugars accumulation in SCZ and YH9

**Fig 3:**
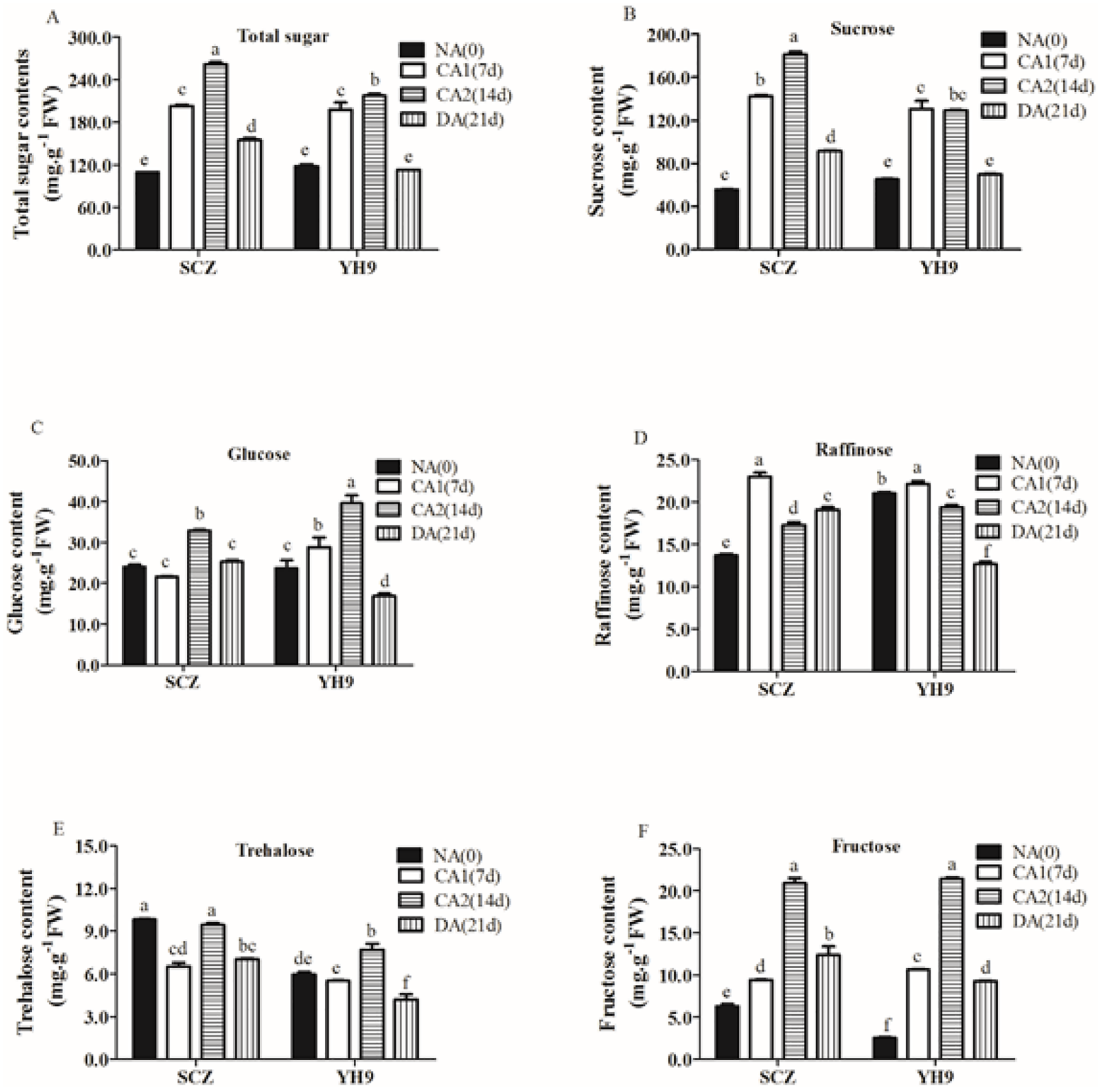
Effects of cold treatment on sugar contents in tea cultivars. Data were displayed as the mean of three replicates and standard error. Columns with different letters had significant differences according to Duncan’s multiple range tests with *P* < 0.05. SCZ and YH9 represent tea cold resistant and cold susceptible tea varieties, respectively. NA: non-acclimation; CA1: cold acclimation of 7 days at 10/4°C, day/night temperature; CA2: cold acclimation of 7 days at 4/0°C, day/night temperature; DA: de-acclimation of 7 days at 25/20°C, day/night temperature.

Sugar accumulation is known to have both osmotic and non-colligative functions, as it stabilizes cell membrane during cold acclimation and enhances freezing resistance in plants [7, 55]. As shown in Fig 3, the total sugar content and the sucrose level in both cultivars were significantly increased under CA condition, with a higher increase observed in SCZ. Relative to NA, the total sugar content in SCZ leaves was increased 2.39-fold after CA2, while the total sugar content in YH9 was increased 1.83-fold after CA2 (Fig 3A). Furthermore, sucrose content in SCZ was increased 2.56-fold after CA1 and reached 3.26-fold after CA2 relative to NA (*P* < 0.05). In contrast, the sucrose content in YH9 leaves was increased 2.0-fold after CA1 and remained constant after CA2 relative to CA1 (Fig 3B). In addition, CA1 and CA2 also induced a moderate increase in glucose and fructose contents in SCZ and YH9 (*P* < 0.05) (Fig 3C and 3F). Differently, CA1 and CA2 induced a little accumulation of raffinose in SCZ (*P* < 0.05), while only a small accumulation of trehalose was observed in YH9 under CA2 (*P* < 0.05) (Fig 3D and 3E). After DA, individual sugar content was decreased by a varying degree (Fig 3).

### Effect of CA and DA on proline accumulation between SCZ and YH9

**Fig 4:**
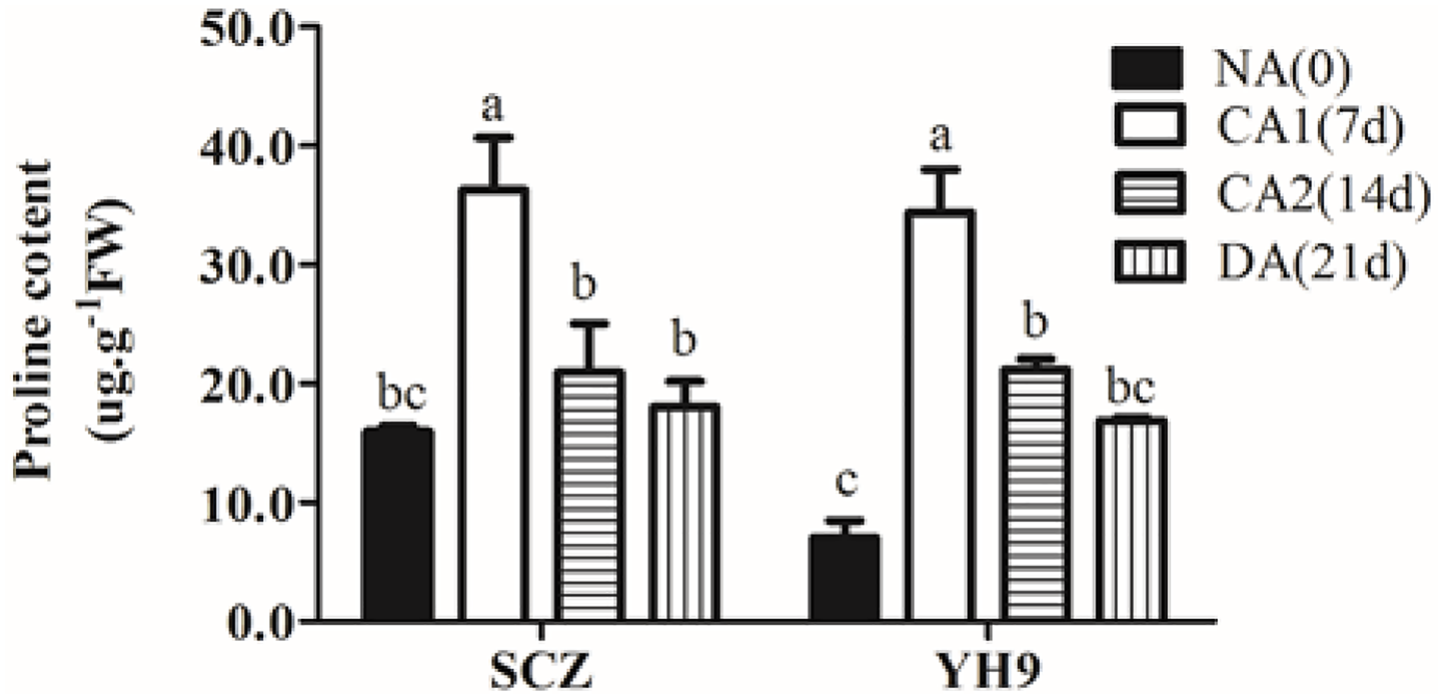
Effects of cold treatment on proline accumulation in tea cultivars. Data are displayed as the mean of three replicates and standard error. Columns with different letters had significant differences according to Duncan’s multiple range tests with *P* < 0.05. SCZ and YH9 represent tea cold resistant and cold susceptible tea varieties, respectively. NA: non-acclimation; CA1: cold acclimation of 7 days at 10/4°C, day/night temperature; CA2: cold acclimation of 7 days at 4/0°C, day/night temperature; DA: de-acclimation of 7 days at 25/20°C, day/night temperature.

As the proline is a multi-functioned osmotic protective substance involved in cold tolerance [39], we measured the changes in proline content in SCZ and YH9 during CA and DA (Fig 4). As shown in Fig 4, the proline levels in both cultivar SCZ and YH9 were increased by 2.27-fold and 4.9-fold during CA1, respectively, and accumulated to similar contents. The study also revealed that the proline content reached the peak in CA1, and afterwards gradually decreased in both SCZ and YH9 (Fig 4).

### Effect of CA and DA on the gene expression of the ICE1-CBF pathway

**Fig 5:**
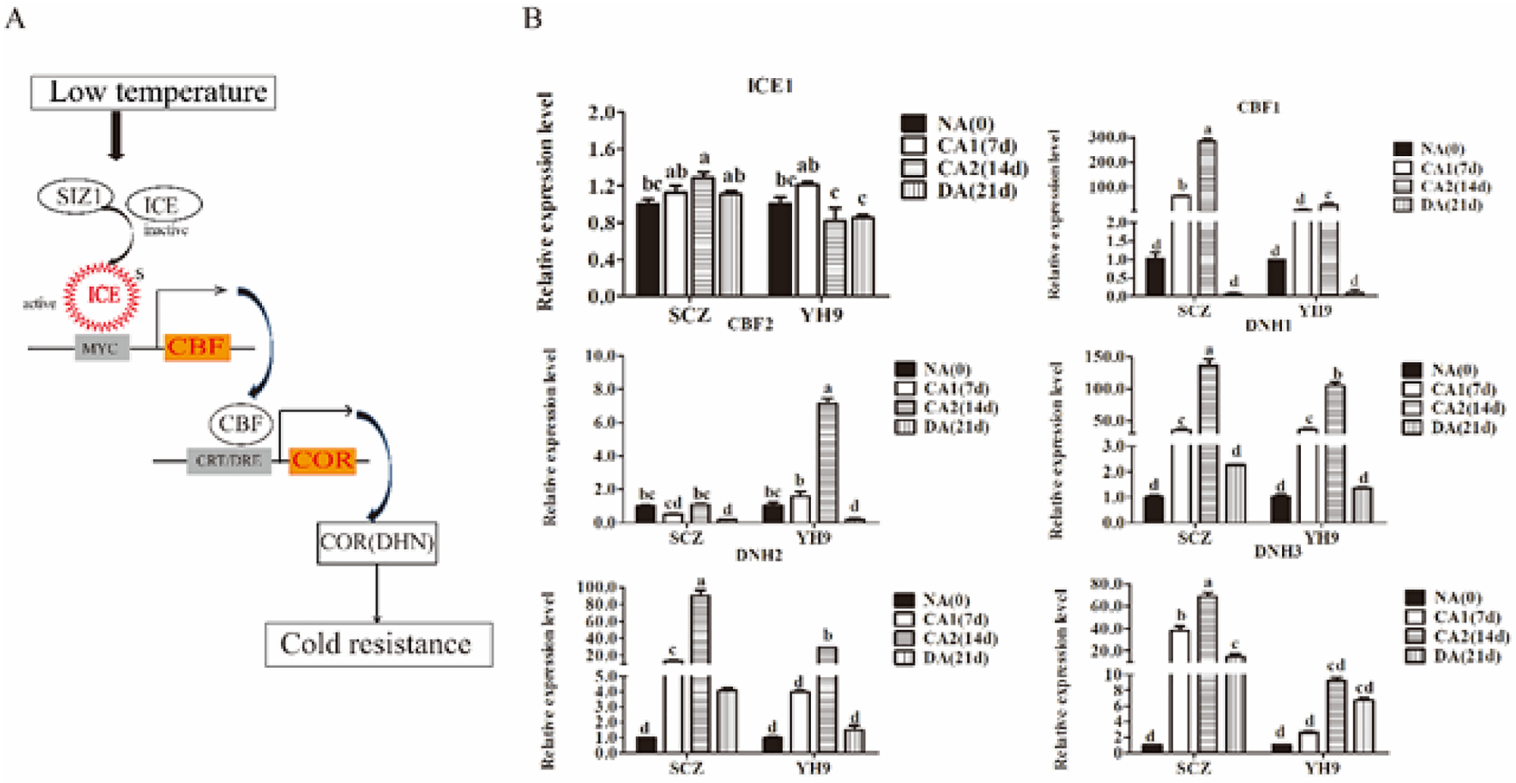
Regulation of the CBF signaling pathway. The pathway (A) was modified from Thomashow, 1999. Relative expression of the genes in ICE-CBF-COR pathway in SCZ and YH9 over CA and DA was shown in B. Gene transcript level was quantified using real-time quantitative RT-PCR approach. GAPDH was used as a control. Data are displayed as the mean of three replicates and standard error. Different letters indicate significant differences to Duncan’s multiple range tests with *P* < 0.05. SCZ and YH9 represent tea cold resistant and cold susceptible tea varieties, respectively. NA: non-acclimation; CA1: cold acclimation of 7 days at 10/4°C, day/night temperature; CA2: cold acclimation of 7 days at 4/0°C, day/night temperature; DA: de-acclimation of 7days at 25/20°C, day/night temperature.

The transcription of *CBFs* is regulated by ICE1 protein, which binds to the DRE/CRT cis-elements in the promoter regions of CORs (Fig 5A). *CBFs* play a central role in integrating the activation of multiple components of the CA respond to chilling and freezing stress in plants [31, 56]. This study did not observe significant changes in *CsICE1* transcription in SCZ and YH9 during CA and DA (Fig 5B). The expression of Cs*CBF1* was significantly increased during CA, and reached approximately 282-fold and 29-fold in CA2, in SCZ and YH9, respectively. The transcript level of *CsCBF1* during CA was 7.1–9.5 folds higher in SCZ than that of YH9 (*P* < 0.05) (Fig 5B). In contrast, the transcript level of *CsCBF2* was increased in YH9 but remained unaffected in SCZ during CA (Fig 5B). For COR genes, the transcription of *CsDNH1*, *CsDNH2* and *CsDNH3* in both cultivars were increased significantly during CA and rapidly decreased following DA. Furthermore, the transcript levels of *DHNs* were higher in SCZ than in YH9 (Fig 5B). Specifically, *CsDNH3* transcript level in SCZ was dramatically increased by 68.7-fold in CA2, while it was only increased by 9.2-fold in YH9 (Fig 5B).

### Effect of CA and DA on the transcription of sucrose- and raffinose-related genes

**Fig 6:**
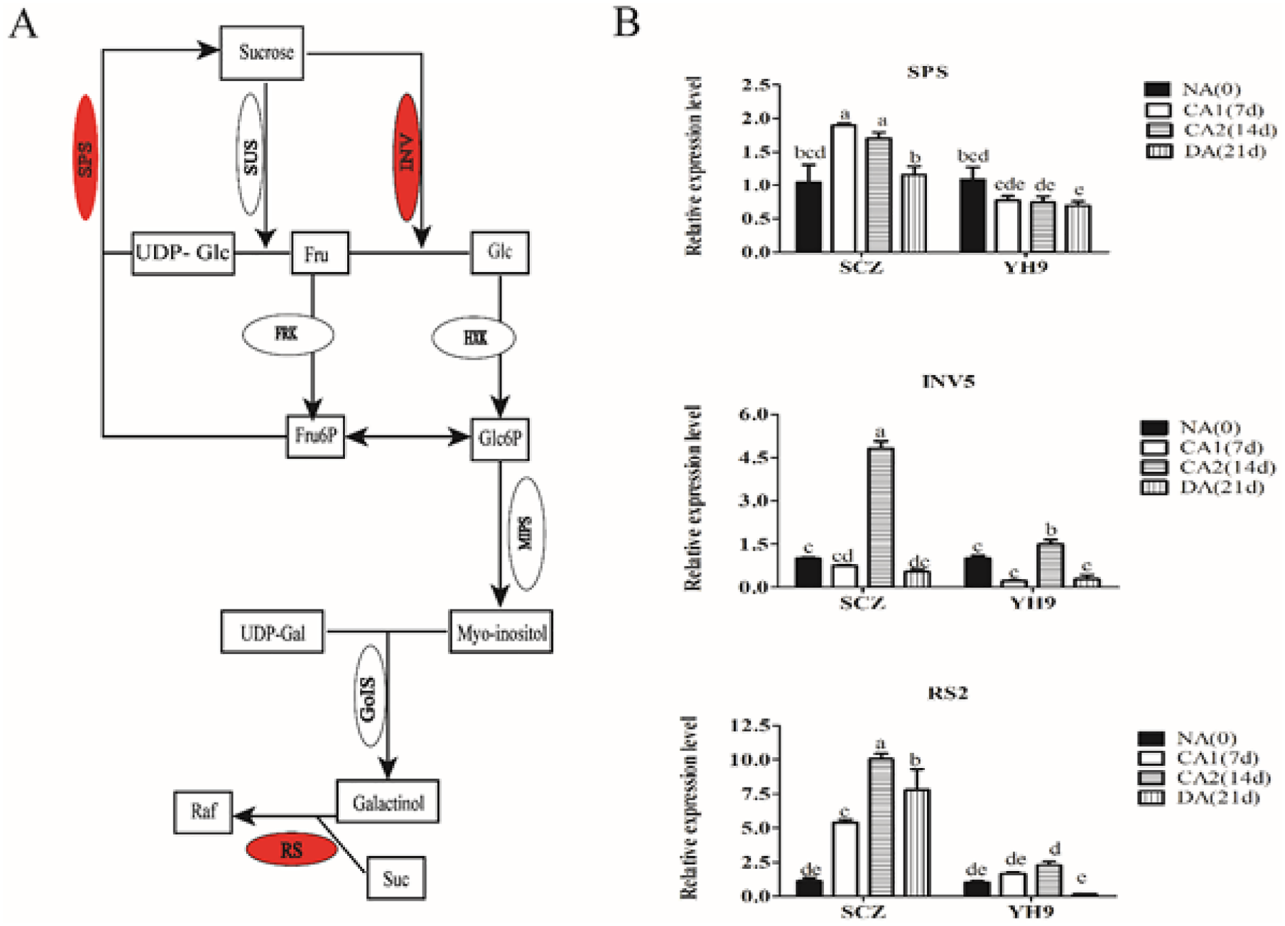
Effect of cold treatment on gene expression of sugar metabolism (Image A, modified from Yue et al, 2015) in tea. Gene transcript level (Image B) was quantified using real-time quantitative RT-PCR approach. GAPDH was used as a control. Data are displayed as the mean of three replicates and standard error. Different letters indicate significant differences to Duncan’s multiple range tests with *P* < 0.05. SCZ and YH9 represent tea cold resistant and cold susceptible tea varieties, respectively. NA: non-acclimation; CA1: cold acclimation of 7 days at 10/4°C, day/night temperature; CA2: cold acclimation of 7 days at 4/0°C, day/night temperature; DA: de-acclimation of 7 days at 25/20°C, day/night temperature.

The *CsSPS* and *CsINV5* are responsible for sucrose accumulation and converting to other saccharides (Fig 6A). The transcripts levels of these two genes were checked in both cultivars during CA and DA. We found that the transcript level of *CsSPS* increased in tea cultivar SCZ but reduced in YH9 through all CA stages, especially in stage CA1 after seven days of CA (Fig 6B). However, *CsINV5* expression was decreased at CA1 after seven days of CA but increased by three folds (*P* < 0.05) at CA2 after 14 days in both cultivars. *CsINV5* expression in cultivar SCZ increased by three folds at CA2 compared with less than one fold in YH9 (Fig 6B). Raffinose synthase gene *CsRS2*, responsible for synthesizing of raffinose, expression was also increased during CA in variety SCZ and then decreased following DA, while it had no distinct changes during CA in YH9 (Fig 6B). This suggested that the genes responsible for sucrose and raffinose accumulation acted positively to regulate the corresponding sugar accumulation in cold treatment.

### Effect of CA and DA on proline biosynthesis and degradation

**Fig 7:**
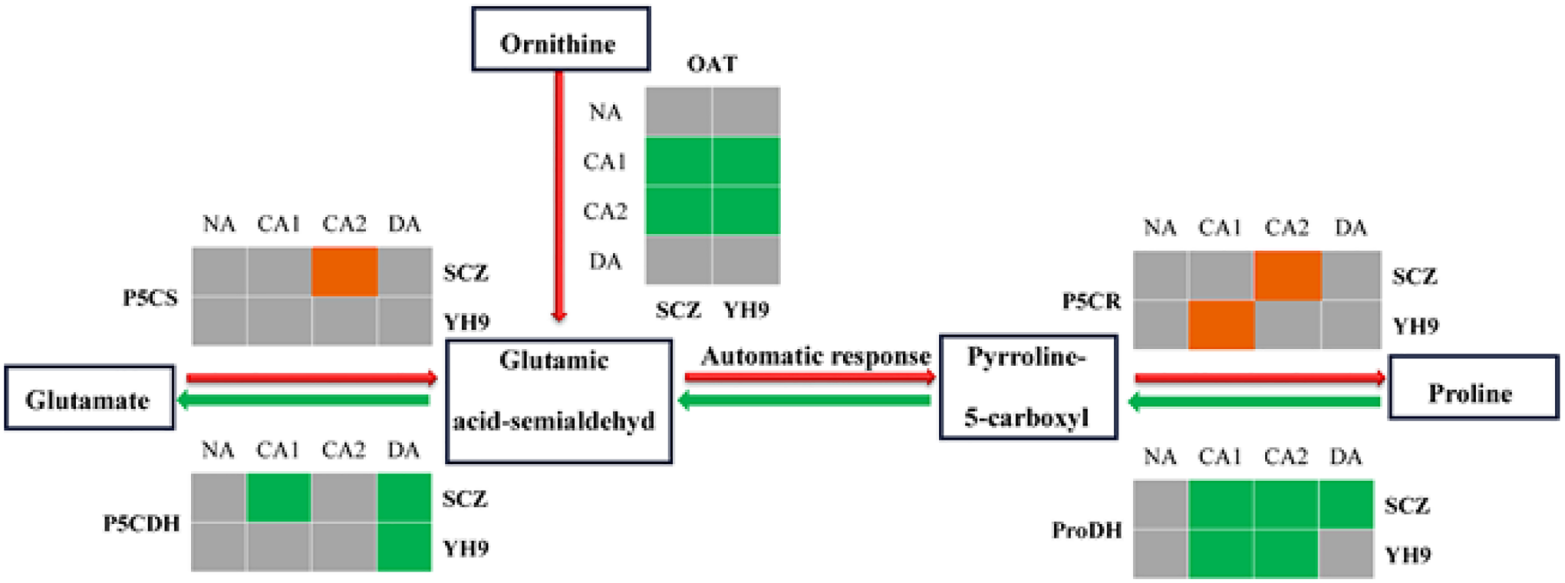
Effect of cold treatment on gene expression in proline-metabolism in tea cultivars. Significant down-, up-regulation and statistically not up/down-regulation is indicated by green color, orange color, and gray color, respectively. Red arrow and green arrow indicate the flow of metabolites with biosynthetic and degradation, respectively. Gene transcript level was quantified using real-time quantitative RT-PCR approach. GAPDH was used as a control. Data are displayed as the mean of three replicates. Significant differences is based on Duncan’s multiple range tests with *P* < 0.05. SCZ and YH9 represent tea cold resistant and cold susceptible tea varieties, respectively. NA: non-acclimation; CA1: cold acclimation of 7 days at 10/4°C, day/night temperature; CA2: cold acclimation of 7 days at 4/0°C, day/night temperature; DA: de-acclimation of 7 days at 25/20°C, day/night temperature.

We illustrated the proline synthesis and degradation in plants in schematic diagram (Fig 7). The expression patterns of these proline-associated enzyme encoding genes were studied in SCZ and YH9 over the treatments of CA and DA. *CsP5CS* and *CsP5CR* were up-regulated at CA2 in SCZ after 14-days cold treatment, and their levels were increased by 1.4-fold and 3.0-fold, respectively. By the contrary, no changes in transcription of *CsP5CS* were observed in YH9 over the treatments of CA and DA. While *CsP5CR* was up-regulated in CA1 in YH9. This suggested that long time induced *CsP5CR* in stage CA2 may be responsible for higher cold tolerance. In both cultivars, the transcription of *CsOAT* was decreased during CA treatments and returned to the normal level after DA treatment. In addition, the transcript level of *CsOAT* in SCZ was similar to that in YH9 (Fig 7). *CsP5CDH* was down-regulated in SCZ during CA1, whereas was unaffected by the CA treatments in YH9 (Fig 7). The transcription of *CsProDH* in both cultivars was decreased during CA treatments (Fig 7).

## Discussion

Low temperature has been a major constraint for tea plantation [47]. During natural CA, tea plants can increase their tolerance to cold weather and survive the winter [8, 45]. In the present study, we demonstrated that plants of either cold-resistant or cold-sensitive tea cultivars can enhance their freezing tolerance due to the treatment of CA in experimental conditions, with a stronger freezing tolerance developed in SCZ than in YH9 (Fig 2B). To understand the mechanisms underlying such differential cold tolerance between SCZ and YH9, we investigated differences between the two cultivars in the physiological and molecular processes that were known to induce cold tolerance in other plants. Our results showed that SCZ exhibited a higher accumulation level of soluble sugars, particularly sucrose than YH9, during cold acclimation (Fig 3). The increased expression of both *CBF1* and its targets *DHNs* could contribute to cold tolerance (Fig 5B). These findings may further elucidate how cold-resistant tea plants can induce strong freezing tolerance in winter.

Through the process of CA, cold resistance was steadily induced in SCZ, while it was induced in YH9 at a slower rate (Fig 2B). Similar to our results, both cold-resistant Medicago (*M*. *falcate*) and cold-susceptible Medicago (*M*. *truncatula*) could enhance their freezing tolerance by CA at 4°C [57]. However, Pennycooke et al. [58] found that the CA-induced freezing tolerance occurred only in cold-resistant plants, but not in cold-susceptible plants. Under cold stress, the inhibition of chlorophyll synthesis and chloroplast formation can lead to reduced Fv/Fm [59]. Bonnecarrère et al. [16] also used the Fv/Fm to identify cold-resistant rice between two japonica genotypes under identical cold stress. Our results indicated that Fv/Fm fell lower in YH9 than in SCZ during CA2 (Fig 2C), suggesting that Fv/Fm, combined with EL50, could be used to evaluate freezing tolerance in cold-resistant and cold-susceptible tea plants.

Soluble sugar accumulation during CA is positively correlated with freezing tolerance in plants [7]. Sucrose was found as a dominant component of enhanced soluble sugars in giant reed and Medicago during CA [57, 60]. But in *Arabidopsis*, the accumulation of glucose is largely responsible for the increased level of soluble sugars during cold acclimation and sucrose is the second most abundant sugar in CA [61]. In our study, the content of sucrose, glucose, raffinose, and fructose were increased during CA in SCZ and YH9, and the total sugar content was higher in SCZ than in YH9 (Fig 3). In other studies, greater accumulation of sucrose in cold resistant Medicago, wheat, and maize was found to be responsible for higher freezing tolerance [57, 62–63]. Trehalose accumulation conferred tolerance to cold stress serving as an osmolyte or protein/membrane protectant by acting as scavengers for ROS to alleviate oxidative damage to the membranes [64]. Therefore, the accumulation of sucrose, as the major sugar, in cold resistant tea plants could play an essential role in conferring higher freezing tolerance in tea plant. Trehalose accumulation was not observed in SCZ during the CA, although the trehalose content was induced slightly in YH9, yet lower than in SCZ (Fig 3E). Our data showed that raffinose contents in the two cultivars were very similar during CA (Fig 3D). However, raffinose was found to be not essential for basic freezing tolerance or for cold acclimation of *A*. *thaliana* [38]. Thus, the role of raffinose in cold resistance in tea plant may be not essential too. Yue et al. [8] reported the content of total sugars and several specific sugars including sucrose, glucose and fructose were constantly elevated in *Longjing43* tea leaves during nature acclimation. While Shen et al. [47] reported the raffinose, maltose, glucose and fructose were all more abundant in HuangShanzhong tea leaves during nature acclimation.

During natural cold acclimation, a series of sugar-related genes, including *CsSPS*, *CsRS2*, and *CsINV5*, are up-regulated in the tea plant (cv. *Longjing43*) [8], which suggests that these genes might be responsible for sugar accumulation. Under the controlled cold treatment and DA in our study, the transcription of the *CsSPS*, *CsINV5*, and *CsRS2* during CA was also up-regulated in SCZ but remained unchanged in YH9 (Fig 6). This demonstrated that the expression regulation of *CsSPS*, *CsINV5*, and *CsRS2* during CA are cultivar specific. Therefore, these genes’ expression can be used as cultivar specific cold resistance indicator for tea breeding.

Free proline has been reported to accumulate in many plants in response to biotic and abiotic stresses, acting as a compatible solute against osmotic stress [39]. Free proline was one of the indicators used to identify dehydration ‘resistant’ wheat genotypes from ‘sensitive’ ones [65]. Kumar and Yadav [66] reported that enhanced proline could increase the tolerance of tea bud to cold stress. Similarly, tea cultivars *‘Zhuyeqi’* (drought-susceptible) and *‘Ningzhou 2*’ (drought-tolerant) could be distinguished due to their differential proline contents under drought stress [67]. Our data showed that proline content was significantly increased in SCZ and YH9 during CA1, but no difference in the total proline content was found between the two cultivars (Fig 4). Therefore, we propose that proline content has effects on the abiotic resistance of tea plants and that the accumulated proline was not a key factor for conferring cold-resistant in tea plants. However, proline concentration is correlated with cold-resistance in giant reed and spring canola [60, 68]. According to Delauney [69], under abiotic stress, proline was accumulated by the glutamate biosynthesis pathway. Our result showed that the *OAT*, responsible for glutamie acid scmialdehyd, transcript level decreased in both cultivars (Fig 7). In accordance with Delauney [69], it was glutamate, not ornithine, which could likely be the main precursor for proline biosynthesis in tea plants during CA. The transcription of *CsP5CS* and *CsP5CR* for proline biosynthesis was higher in SCZ than in YH9, and the transcripts of *CsProDH* and *CsP5CDH* for proline degradation differed between two cultivars (Fig 7). It showed that the transcription level of the related genes was not consistent with metabolic changes and further enzymatic assays are required to elucidate the proline biosynthesis mechanisms.

To date, ICE and CBF genes are known to play key roles in cold tolerance. The transcription of *CsCBF1*, *not CsICE1*, was induced at 4°C [34]. With CA treatment, our results consistently showed that there was not change in expression of *CsICE1* in both cold resistant and susceptible tea cultivars. We found that the transcription of *CsCBF1* was significantly up-regulated by CA, and remained high level until DA (Fig 5B). A higher expression change of *CsCBF1* was found in cold resistant cultivar SCZ than cold susceptible YH9 (Fig 5B), which may explain the difference in cold resistance in the two tea cultivars. A similar result was also found in Medicago and Jatropha [57, 60]. However, Pan et al. [70] reported a contradictory finding that the cold-susceptible rice had a much strong transcription of *CBFs* than cold-resistant rice. This might be caused by the species difference. Our data found a higher up-regulation of *CsCBF2* transcription in YH9 than SCZ during CA (Fig 5B). *AtCBF2* is a negative regulator of *AtCBF1* in *Arabidopsis*. We hypothesized that that *CsCBF2* in tea plant might also be a negative regulator of *CsCBF1*. In this case, the lowered transcription of *CsCBF1* can be explained by the suppression of the increased transcription of *CsCBF2* in YH9. The high level of *CsCBF1* transcription in SCZ was a result of low transcription of *CsCBF2*. Further investigation of the suppression would be the priority in future study. In addition, *CsCBF1* in SCZ and YH9 had the same ORF (Open Reading Frame) without Intron. We speculate that SNP and INDEL may be present in the promoter region of *CsCBF1*, and regulate the transcription of *CsCBF1* between resistant and susceptible species. *DHNs* (COR genes), a subgroup of the late embryogenesis abundant protein family, can act as a cryoprotectant and molecular chaperone as well as an anti-oxidant. *DHNs* can be induced by cold stress, and their transcription is correlated with freezing tolerance [36, 71]. In this study, the transcripts of *CsDHN1*, *CsDHN2*, and *CsDHN3* were accumulated at higher levels in SCZ than YH9 during CA (Fig 5B). Similar results were found in Loquat (Eriobotrya japonica), where seven dehydration genes were up-regulated under low-temperature stress, with significantly higher transcription observed in cold resistance than cold-susceptible cultivars [71]. The higher levels of *CsDHN1*, *CsDHN2*and *CsDHN3* may result in higher amount of dehydrin proteins, thus protecting SCZ from dehydration under freezing stress.

Of course, other mechanisms may involve in the cold acclimation in tea plant. One of them is that the PLD (Phospholipase D) pathway, which responses to freezing and plays key roles in conferring higher cold resistance [11]. PLDs, lipid catabolism enzymes, are activated by a fall in temperature, and the expression levels are found to increase during cold stress [72, 73]. Phosphatidic acid (PA), a catalyzed production of phospholipase D (PLD), involves in many cellular processes, including cell signaling, vesicular trafficking and membrane remodeling [12, 72]. Cold acclimation also affects cell lipid composition, which in favor of the maintenance of plasma membrane functionality and fluidity [10, 74]. In particular, the proportion of unsaturated fatty acids making up the phospholipids is increased [74]. A substantial increase in linoleic acid (C18:2) has been reported for cold acclimated Solanum commersonii plants, a potato wild species able to increase freezing tolerance. While the freezing susceptible species, Solanum tuberosum, was an increase of C18:3 [75]. Fatty acids unsaturation is controlled by a transcriptional regulation of key desaturase genes. The cotyledons of cold acclimated plants produced a high-fold increase in delta 12 desaturase FAD2-3 (FAD2-3) expression compared with non-acclimated plants [13]. For tea plant (Cs var. sinensis), both CsFAD7 and CsFAD8 were cloned, and CsFAD8 genes has a high expression in cold resistant cultivar than susceptible cultivar [48]. Due to a fact that the cold acclimation may not apply to some plants such as crop wheat, it is worthy to investigate how the lipid metabolism is regulated under cold in tea and whether it is correlated with expression level of fatty acid related genes in cold in our future study using RNA-seq and metabolomics strategy.

In summary, the data presented here have demonstrated the difference of physiological, biochemical, and gene expression levels explained the difference in cold tolerance in cold-resistant tea cultivar SCZ and cold-susceptible tea cultivar YH9. These findings have contributed a better insight into the molecular mechanisms that underly cold tolerance in tea plants.

## Supporting information

S1 Text Melt curve and Melt peak of *genes*. (DOCX)

S2 Text Comparative sequence analysis among GenBank accession and qPCR products of SCZ and YH9 of *genes*. (DOCX)

S3 Text Effects of CA on freezing tolerance of SCZ. (DOCX)

## Abbreviations

CA: cold acclimation
CBF: *C-repeat/dehydration-responsive element binding factor*
CsINV5: *Invertase gene*
CsOAT: *Ornithine-D-aminotransferase*
CsP5CDH: *P5Cdehydrogenase*
CsP5CR: *1-pyrroline-5-carboxylate reductase*
CsP5CS: *Δ -1-Pyrroline-5-carboxylate synthase*
CsProDH: *Proline dehydrogenase*
CsRS2: *Raffinose synthase gene*
CsSPS: *Sucrose phosphate synthase*
DA: de-acclimation
DHN: *Dehydrin*
EL50: Temperature leading to 50% tissue damages due to leakage of electrolyte
Fv/Fm: maximum quantum yield of PSII photosystems
SCZ: *Camellia sinensis* cv. *Shuchazao*
YH9: *Camellia sinensis* var. *assamica* cv.*Yinghong9*
