# Supplementary material for: Comparative analysis of the response and gene regulation in cold resistant and susceptible tea plants
Source: PLoS One. 2017 Dec 6;12(12):e0188514. doi: 10.1371/journal.pone.0188514 (PMC5718485; doi:10.1371/journal.pone.0188514)
Supplement: S1 Text — (DOCX) [file pone.0188514.s001.docx]

**S1 Text. Melt curve and Melt Peak of *genes***

**S1 Text Contents**

**S1 Text. Figure A. Melt curve and Melt Peak of *GAPDH***

**S1 Text. Figure B. Melt curve and Melt Peak of *CsICE1***

**S1 Text. Figure C. Melt curve and Melt Peak of *CsCBF1***

**S1 Text. Figure D. Melt curve and Melt Peak of *CsCBF2***

**S1 Text. Figure E. Melt curve and Melt Peak of *CsDNH1***

**S1 Text. Figure F. Melt curve and Melt Peak of *CsDNH2***

**S1 Text. Figure G. Melt curve and Melt Peak of *CsDNH3***

**S1 Text. Figure H. Melt curve and Melt Peak of *CsSPS***

**S1 Text. Figure I. Melt curve and Melt Peak of *CsINV5***

**S1 Text. Figure J. Melt curve and Melt Peak of *CsRS2***

**S1 Text. Figure K. Melt curve and Melt Peak of *CsP5CS***

**S1 Text. Figure L. Melt curve and Melt Peak of *CsOAT***

**S1 Text. Figure M. Melt curve and Melt Peak of *CsP5CDH***

**S1 Text. Figure N. Melt curve and Melt Peak of *CsP5CR***

**S1 Text. Figure O. Melt curve and Melt Peak of *CsPRODH***


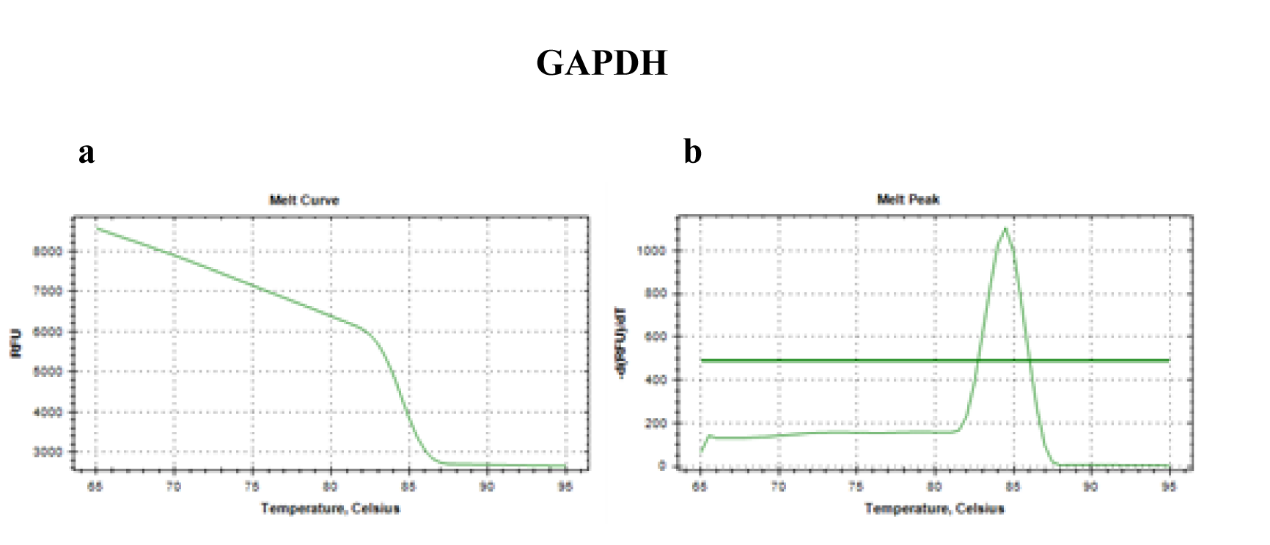


**S1. Figure A. Melt curve and Melt Peak of *GAPDH*** Image a is melt curve of *GAPDH* and Image b is melt peak of *GAPDH.*


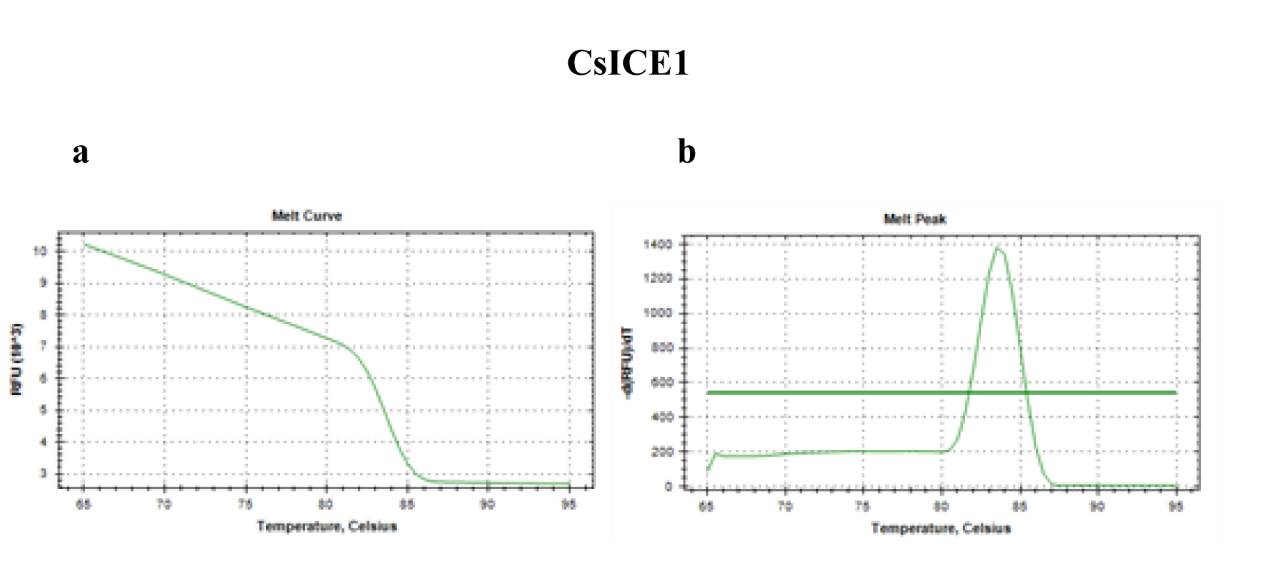


**S1. Figure B. Melt curve and Melt Peak of *CsICE1*** Image a is melt curve of *CsICE1*and Image b is melt peak of *CsICE1*.


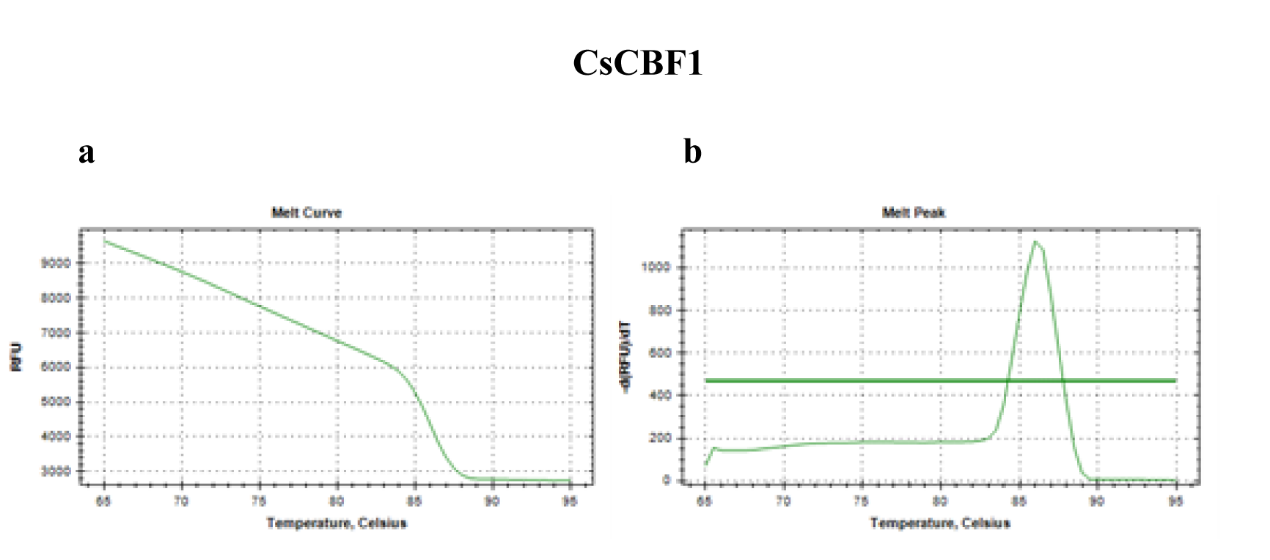


**S1. Figure C. Melt curve and Melt Peak of *CsCBF1*** Image a is melt curve of *CsCBF1* and Image b is melt peak of *CsCBF1*.


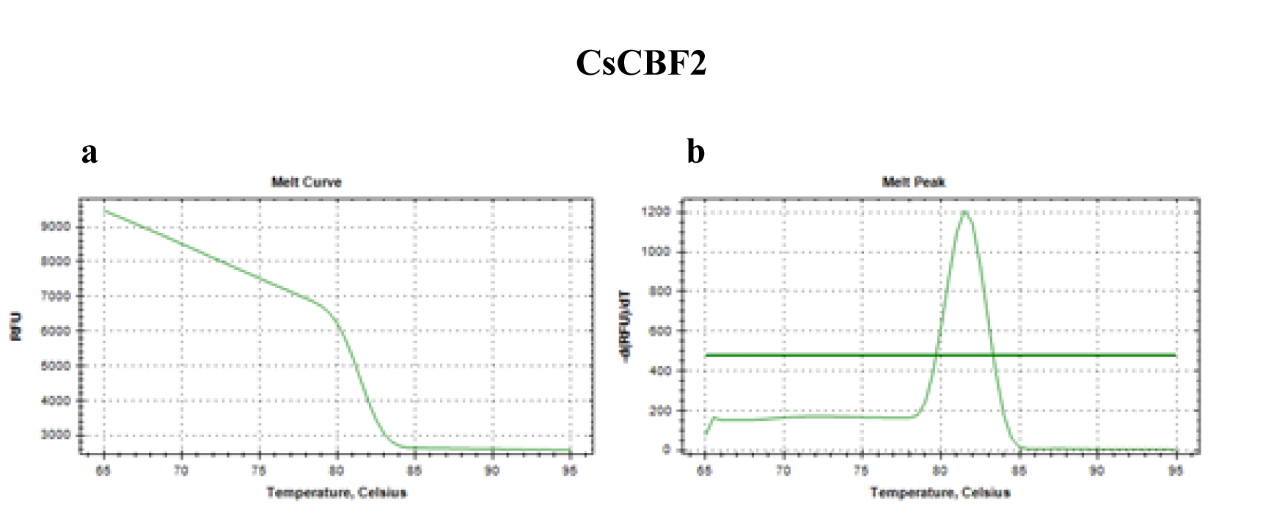


**S1. Figure D. Melt curve and Melt Peak of *CsCBF2*** Image a is melt curve of *CsCBF2* and Image b is melt peak of *CsCBF2*.


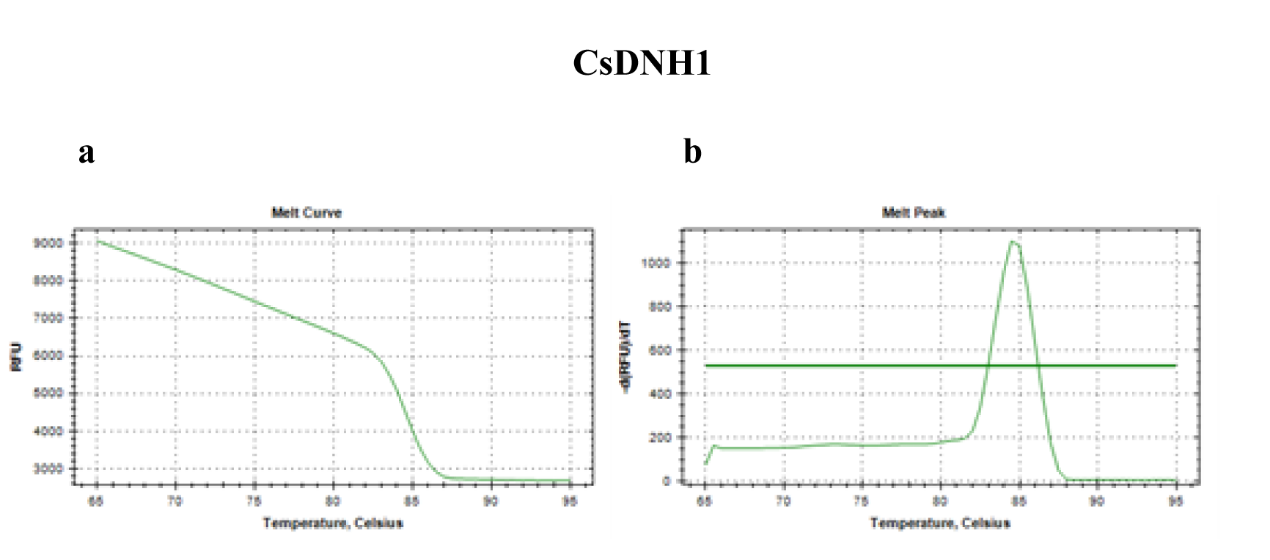


**S1. Figure E. Melt curve and Melt Peak of *CsDNH1*** Image a is melt curve of *CsDNH1* and Image b is melt peak of *CsDNH1*.


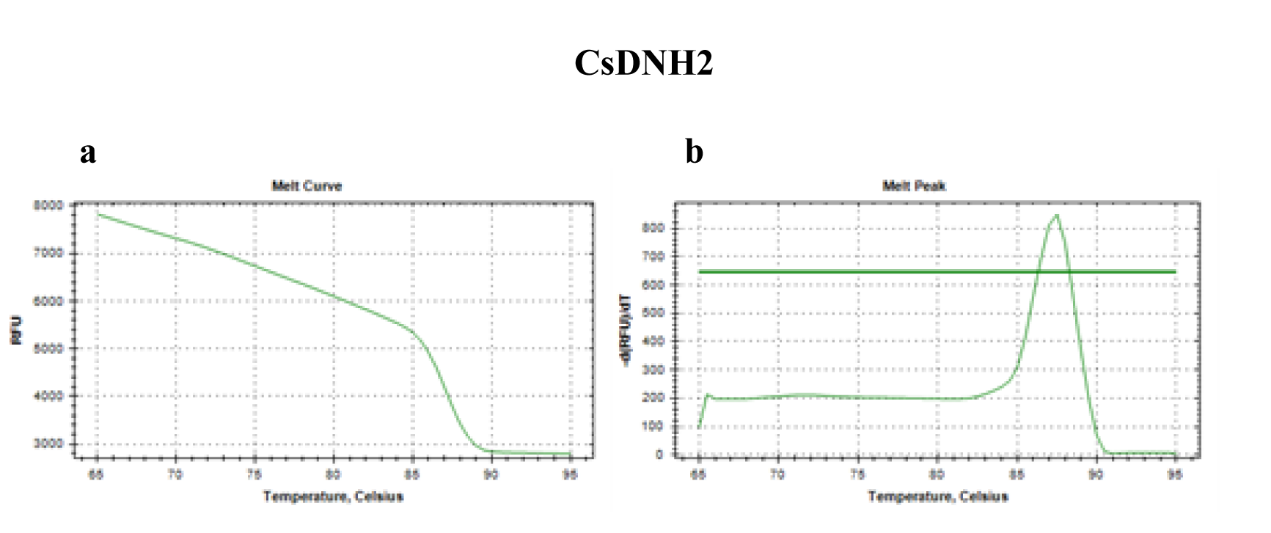


**S1. Figure F. Melt curve and Melt Peak of *CsDNH2*** Image a is melt curve of *CsDNH2* and Image b is melt peak of *CsDNH2*.


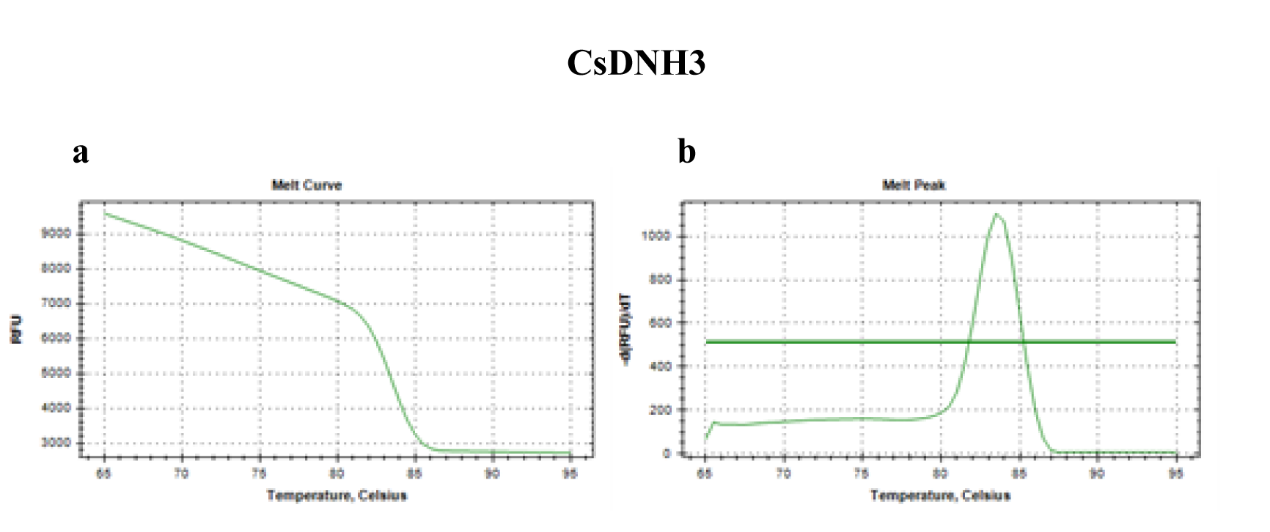


**S1. Figure G. Melt curve and Melt Peak of *CsDNH3*** Image a is melt curve of *CsDNH3* and Image b is melt peak of *CsDNH3*.


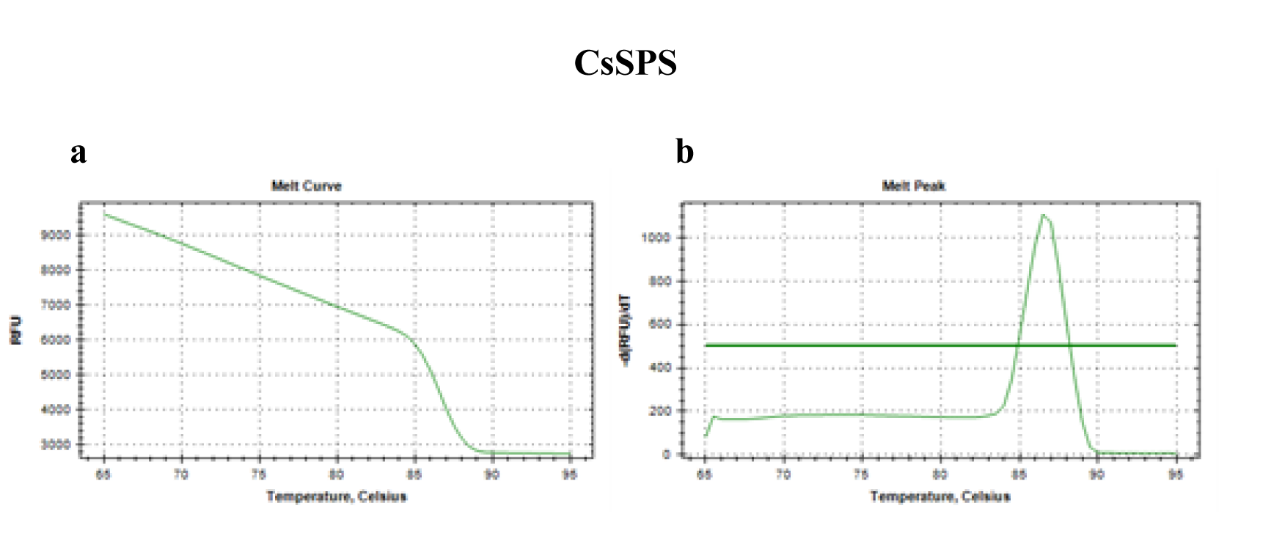


**S1. Figure H. Melt curve and Melt Peak of *CsSPS*** Image a is melt curve of *CsSPS* and Image b is melt peak of *CsSPS*.


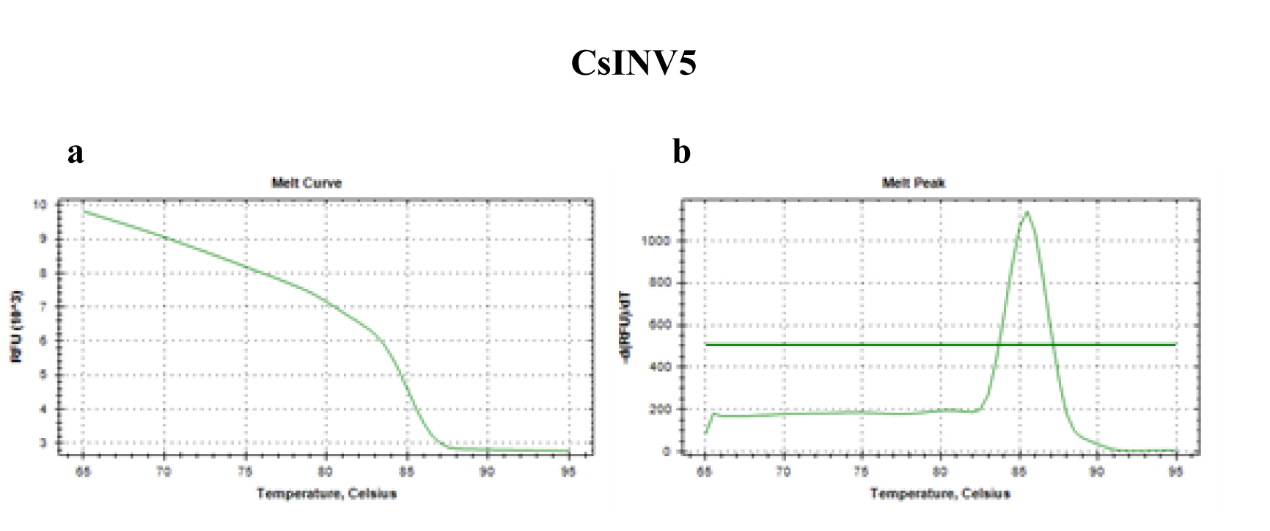


**S1. Figure I. Melt curve and Melt Peak of *CsINV5*** Image a is melt curve of *CsINV5* and Image b is melt peak of *CsINV5*.


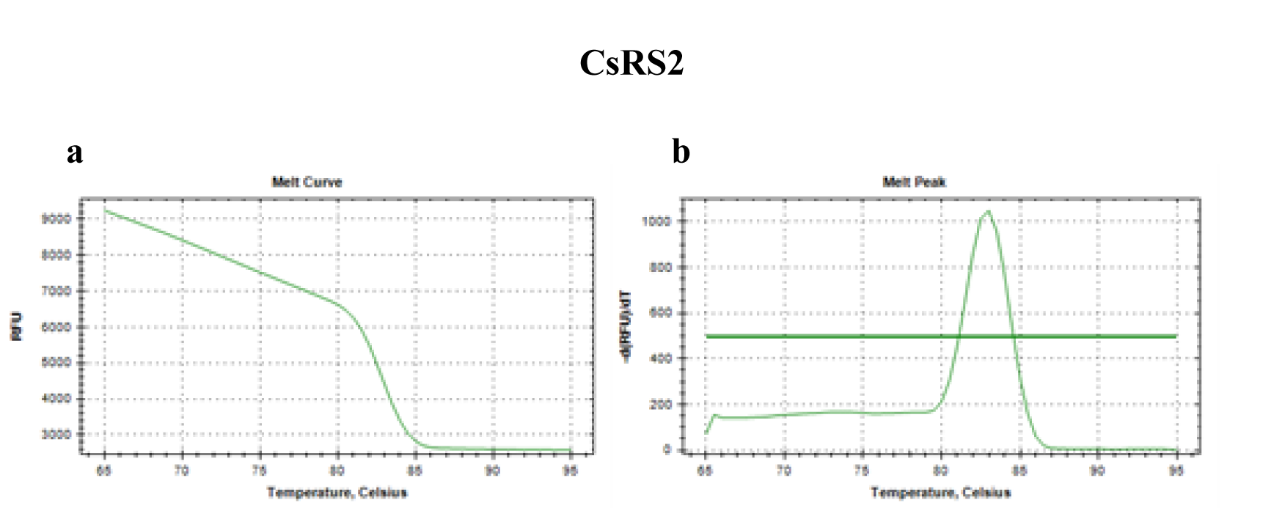


**S1. Figure J. Melt curve and Melt Peak of *CsRS2*** Image a is melt curve of *CsRS2* and Image b is melt peak of *CsRS2.*


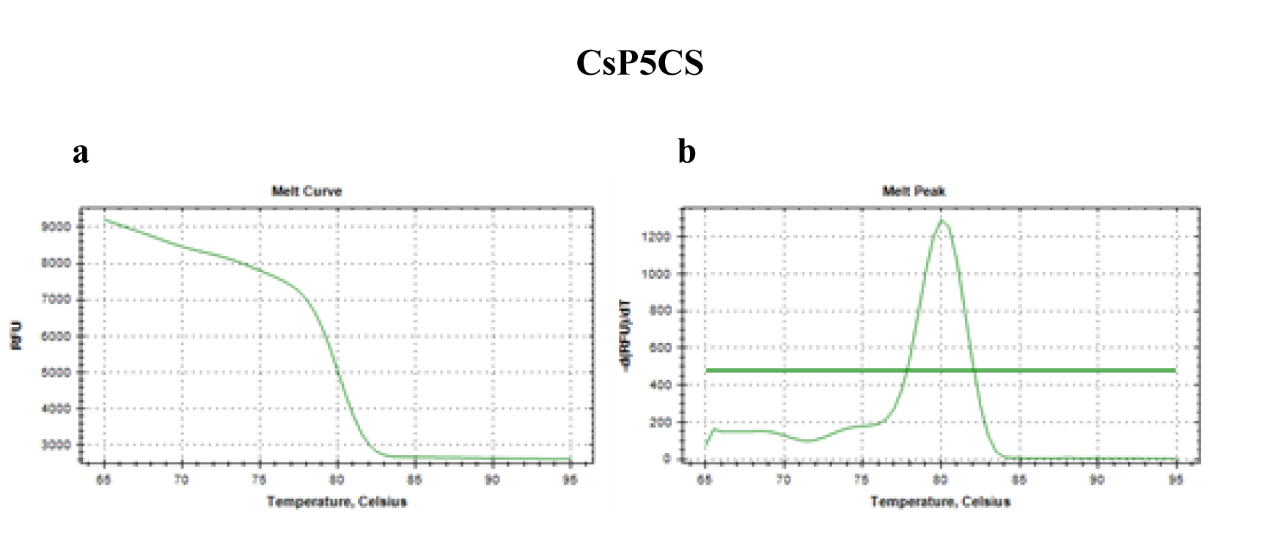


**S1. Figure K. Melt curve and Melt Peak of *CsP5CS*** Image a is melt curve of *CsP5CS* and Image b is melt peak of *CsP5CS*.


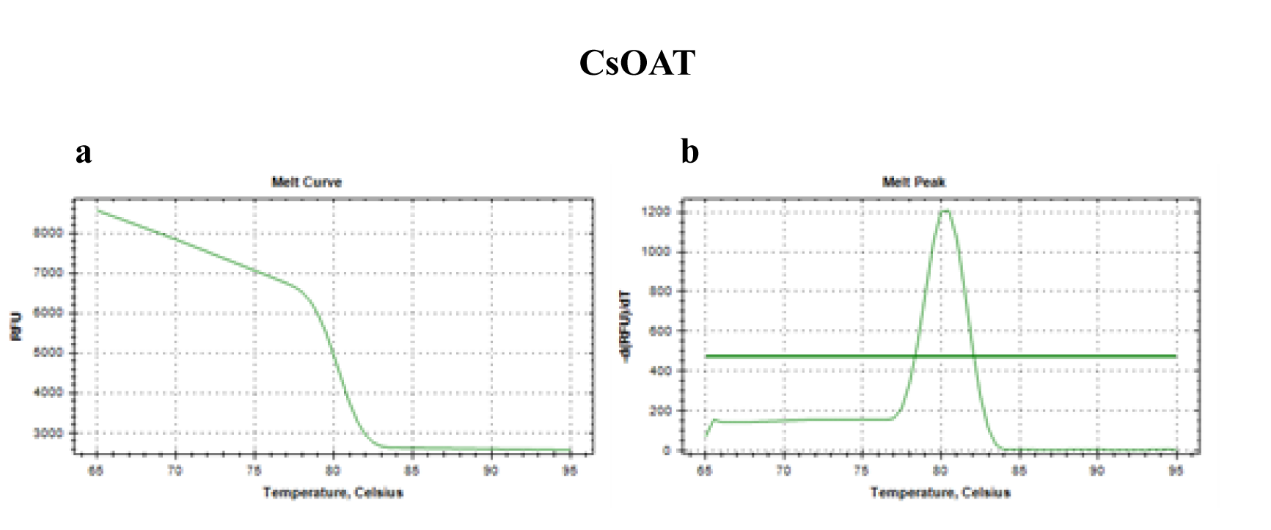


**S1. Figure L. Melt curve and Melt Peak of *CsOAT*** Image a is melt curve of *CsOAT* and Image b is melt peak of *CsOAT*.


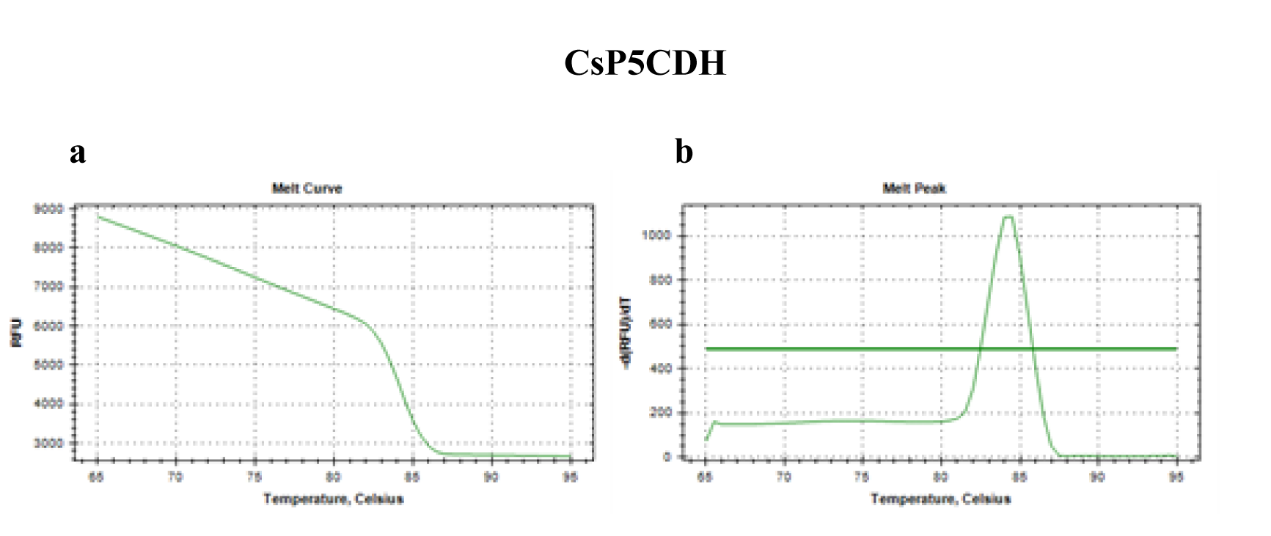


**S1. Figure M. Melt curve and Melt Peak of *CsP5CDH*** Image a is melt curve of *CsP5CDH* and Image b is melt peak of *CsP5CDH*.


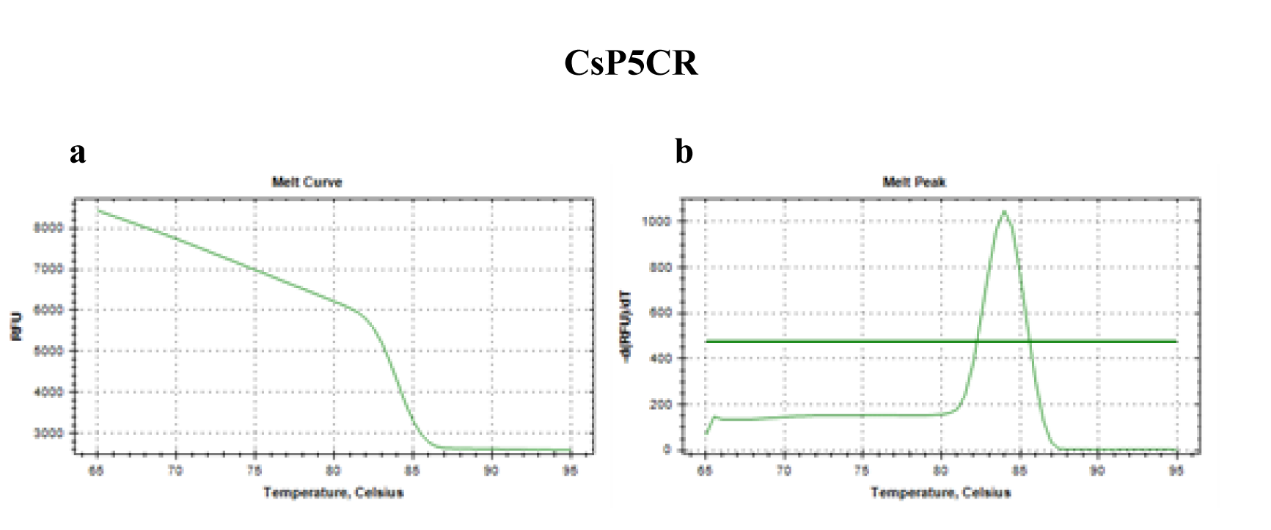


**S1. Figure N. Melt curve and Melt Peak of *CsP5CR*** Image a is melt curve of *CsP5CR* and Image b is melt peak of *CsP5CR*.


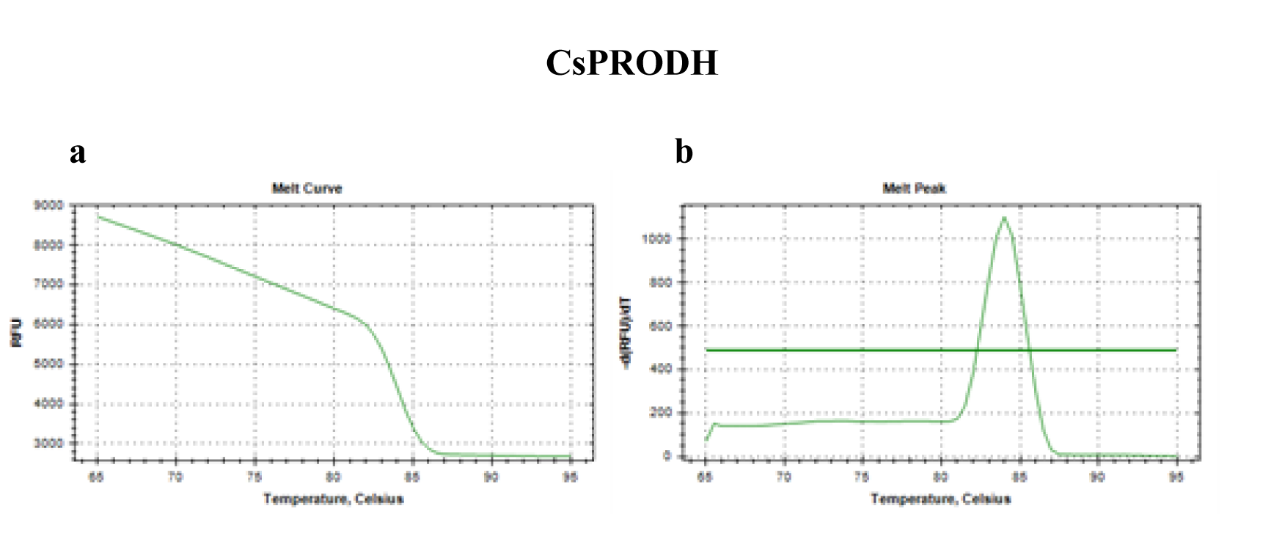


**S1. Figure O. Melt curve and Melt Peak of *CsPRODH*** Image a is melt curve of *CsPRODH* and Image b is melt peak of *CsPRODH*.
