# Supplementary material for: Comparative analysis of the response and gene regulation in cold resistant and susceptible tea plants
Source: PLoS One. 2017 Dec 6;12(12):e0188514. doi: 10.1371/journal.pone.0188514 (PMC5718485; doi:10.1371/journal.pone.0188514)
Supplement: S2 Text — (DOCX) [file pone.0188514.s002.docx]

**S2 Text. Comparative sequence analysis among GenBank Accession and qPCR products of SCZ and YH9 of** ***genes***

**S2 Text Contents**

**S2 Text. Figure A. Comparative sequence analysis among GenBank Accession and qPCR products of SCZ and YH9 of *GAPDH***

**S2 Text. Figure B. Comparative sequence analysis among GenBank Accession and qPCR products of SCZ and YH9 of *CsICE1***

**S2 Text. Figure C. Comparative sequence analysis among GenBank Accession and qPCR products of SCZ and YH9 of *CsCBF1***

**S2 Text. Figure D. Comparative sequence analysis among GenBank Accession and qPCR products of SCZ and YH9 of *CsCBF2***

**S2 Text. Figure E. Comparative sequence analysis among GenBank Accession and qPCR products of SCZ and YH9 of *CsDNH1***

**S2 Text. Figure F. Comparative sequence analysis among GenBank Accession and qPCR products of SCZ and YH9 of *CsDNH2***

**S2 Text. Figure G. Comparative sequence analysis among GenBank Accession and qPCR products of SCZ and YH9 of *CsDNH3***

**S2 Text. Figure H. Comparative sequence analysis among GenBank Accession and qPCR products of SCZ and YH9 of *CsSPS***

**S2 Text. Figure I. Comparative sequence analysis among GenBank Accession and qPCR products of SCZ and YH9 of *CsINV5***

**S2 Text. Figure J. Comparative sequence analysis among GenBank Accession and qPCR products of SCZ and YH9 of *CsRS2***

**S2 Text. Figure K. Comparative sequence analysis among GenBank Accession and qPCR products of SCZ and YH9 of *CsP5CS***

**S2 Text. Figure L. Comparative sequence analysis among GenBank Accession and qPCR products of SCZ and YH9 of *CsOAT***

**S2 Text. Figure M. Comparative sequence analysis among GenBank Accession and qPCR products of SCZ and YH9 of *CsP5CDH***

**S2 Text. Figure N. Comparative sequence analysis among GenBank Accession and qPCR products of SCZ and YH9 of *CsP5CR***

**S2 Text. Figure O. Comparative sequence analysis among GenBank Accession and qPCR products of SCZ and YH9 of *CsPRODH***


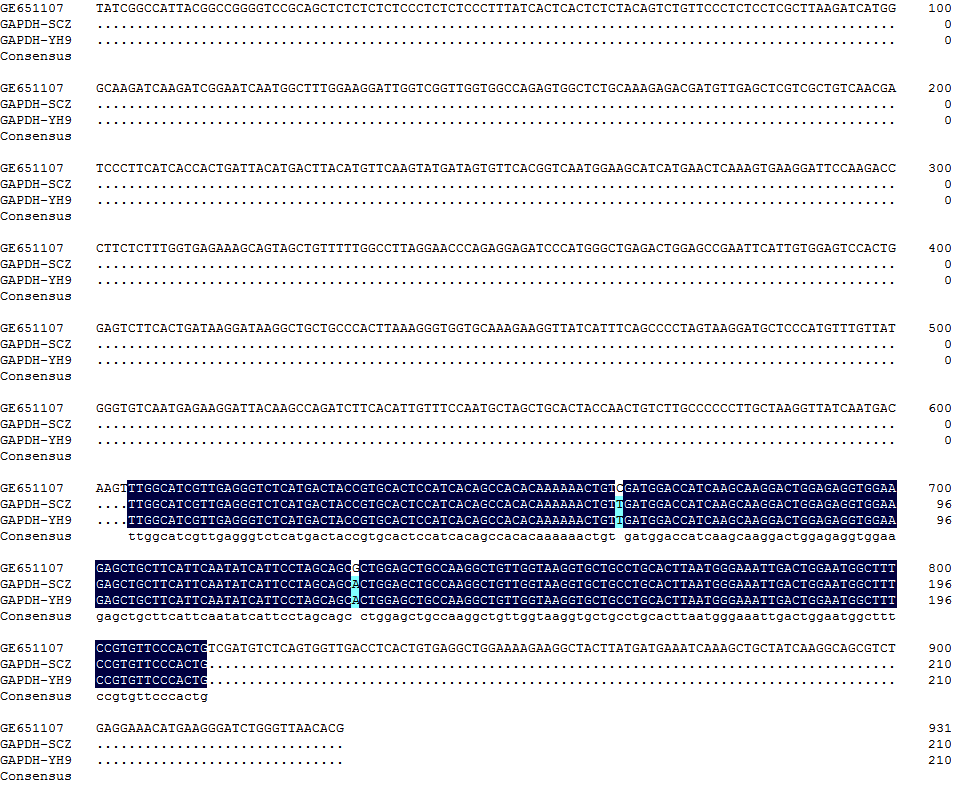


**S2. Figure A. Comparative sequence analysis among GenBank Accession and qPCR products of SCZ and YH9 of *GAPDH*.** Identical base sequences are shaded blue.

**
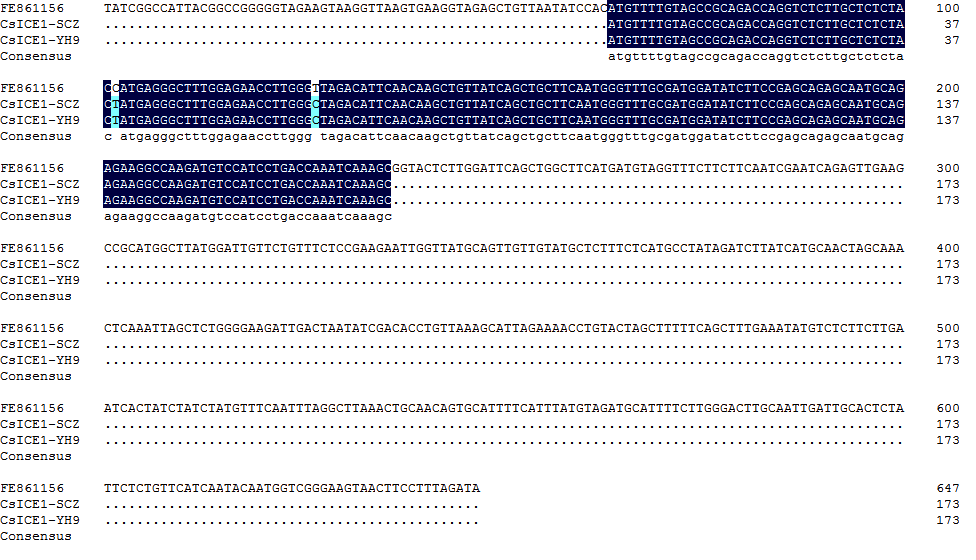
**

**S2. Figure B. Comparative sequence analysis among GenBank Accession and qPCR products among SCZ and YH9 of *CsICE2*.** Identical base sequences are shaded blue.

**
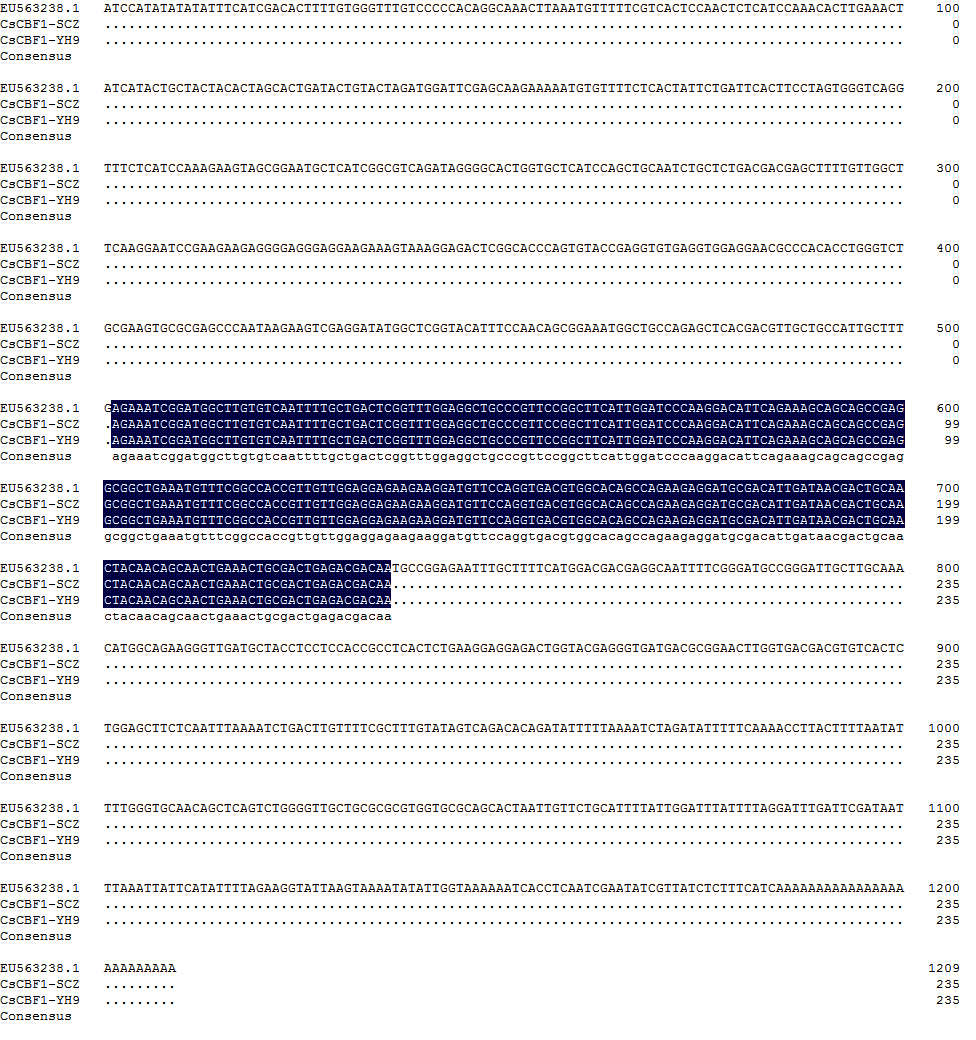
**

**S2. Figure C. Comparative sequence analysis among GenBank Accession and qPCR products of SCZ and YH9 of *CsCBF2*.** Identical base sequences are shaded blue.

**
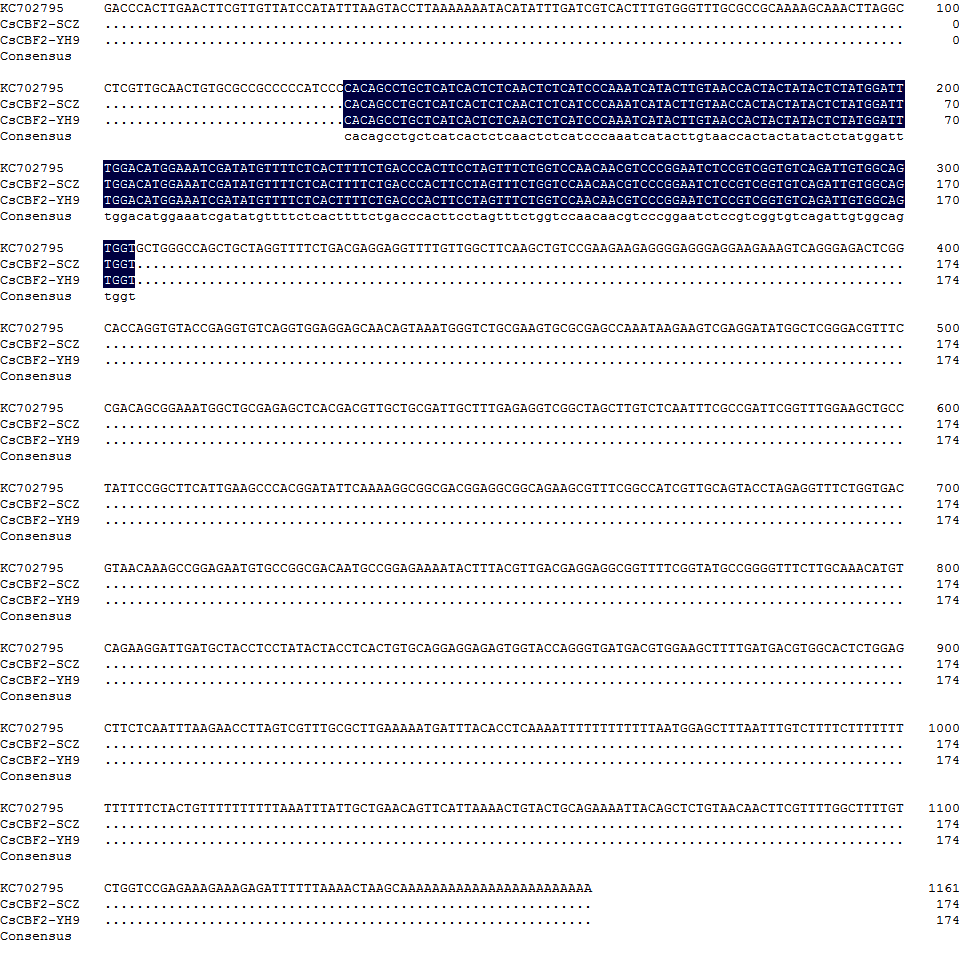
**

**S2. Figure D. Comparative sequence analysis among GenBank Accession and qPCR products of SCZ and YH9 of *CsCBF2.*** Identical base sequences are shaded blue.

**
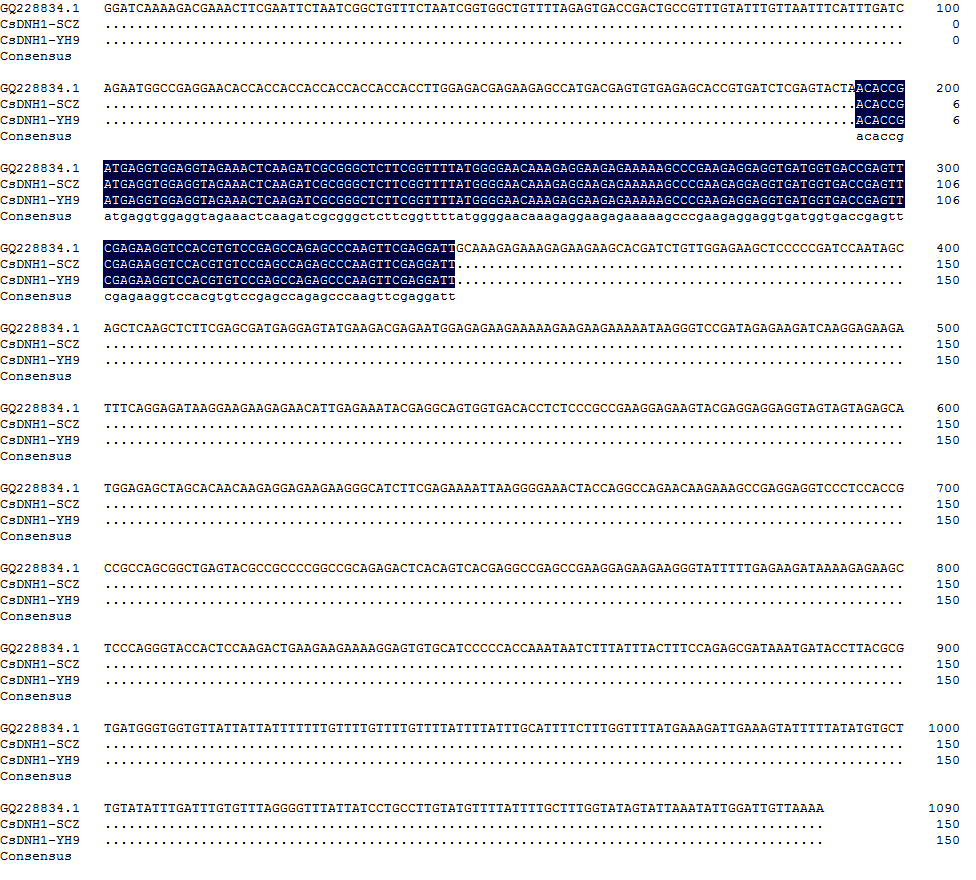
**

**S2. Figure E. Comparative sequence analysis among GenBank Accession and qPCR products of SCZ and YH9 of *CsDNH2*.** Identical base sequences are shaded blue.

**
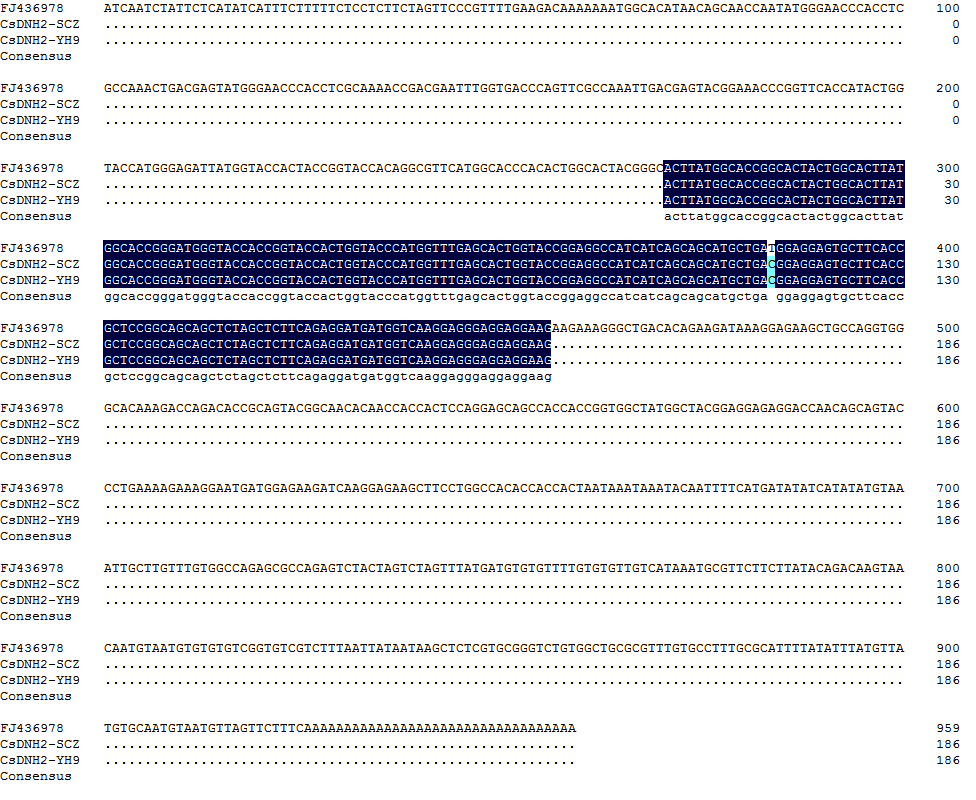
**

**S2. Figure F. Comparative sequence analysis among GenBank Accession and qPCR products of SCZ and YH9 of *CsDNH2*.** Identical base sequences are shaded blue.

**
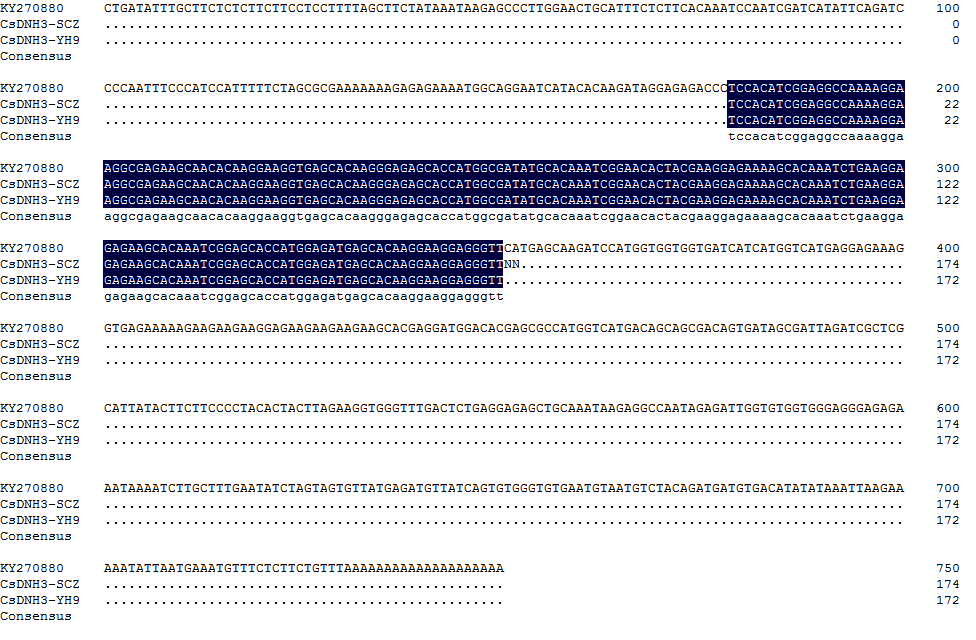
**

**S2. Figure G. Comparative sequence analysis among GenBank Accession and qPCR products of SCZ and YH9 of *CsDNH3.*** Identical base sequences are shaded blue.

**
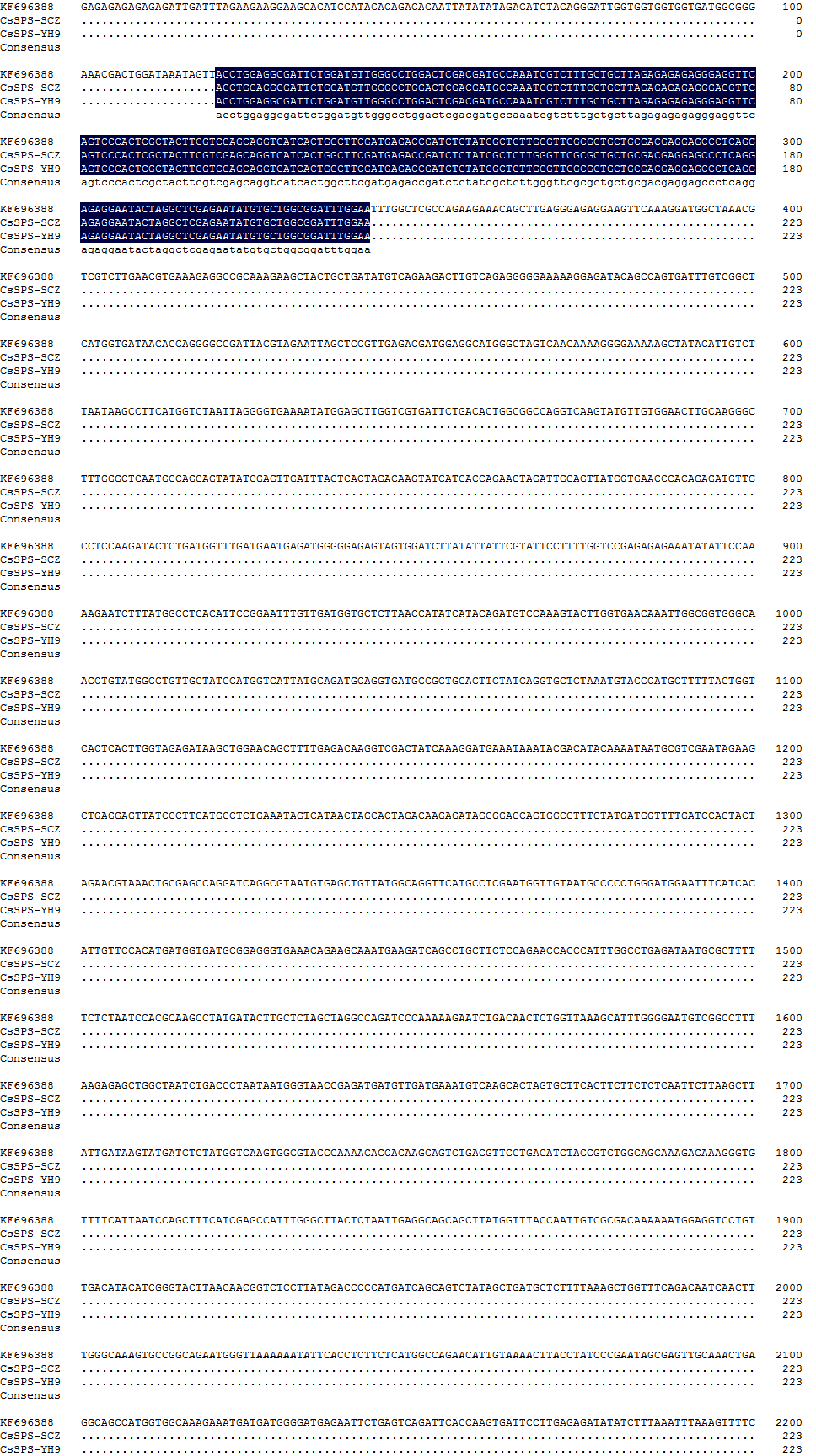
**

**S2. Figure H. Comparative sequence analysis among GenBank Accession and qPCR products of SCZ and YH9 of *CsSPS*.** Identical base sequences are shaded blue.

**
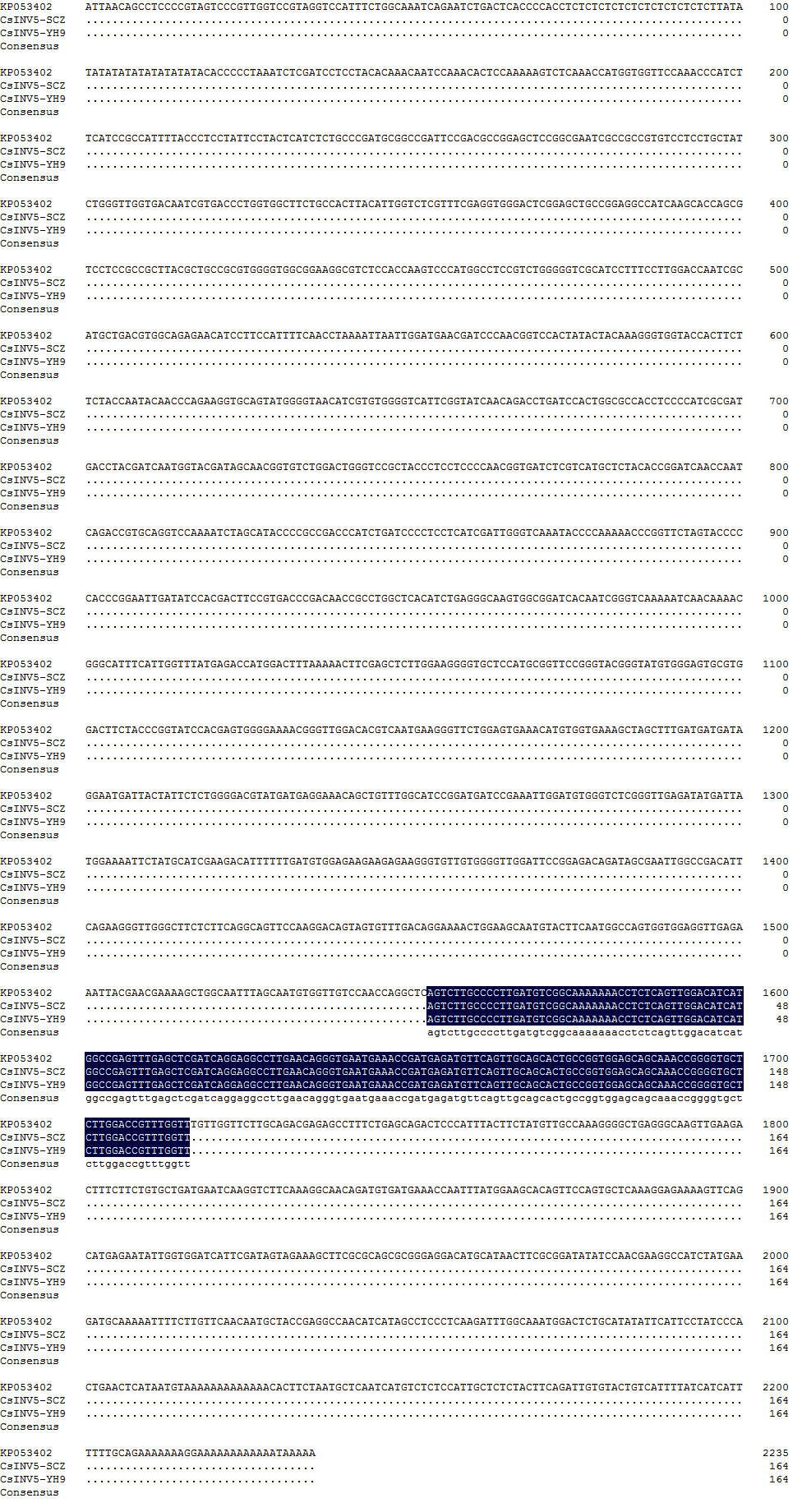
**

**S2. Figure I. Comparative sequence analysis among GenBank Accession and qPCR products of SCZ and YH9 of *CsINV5*.** Identical base sequences are shaded blue.

**
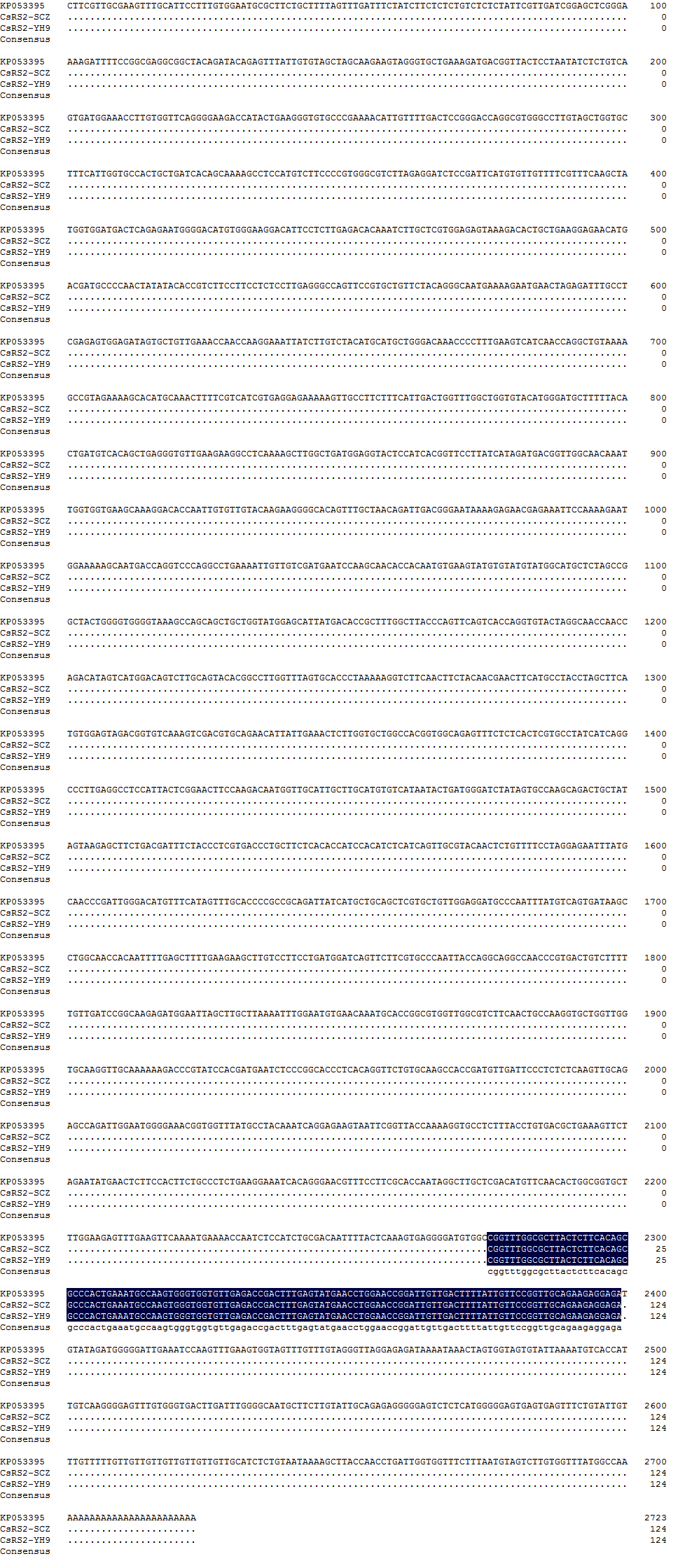
**

**S2. Figure J. Comparative sequence analysis among GenBank Accession and qPCR products of SCZ and YH9 of *CsRS2*.** Identical base sequences are shaded blue.

**
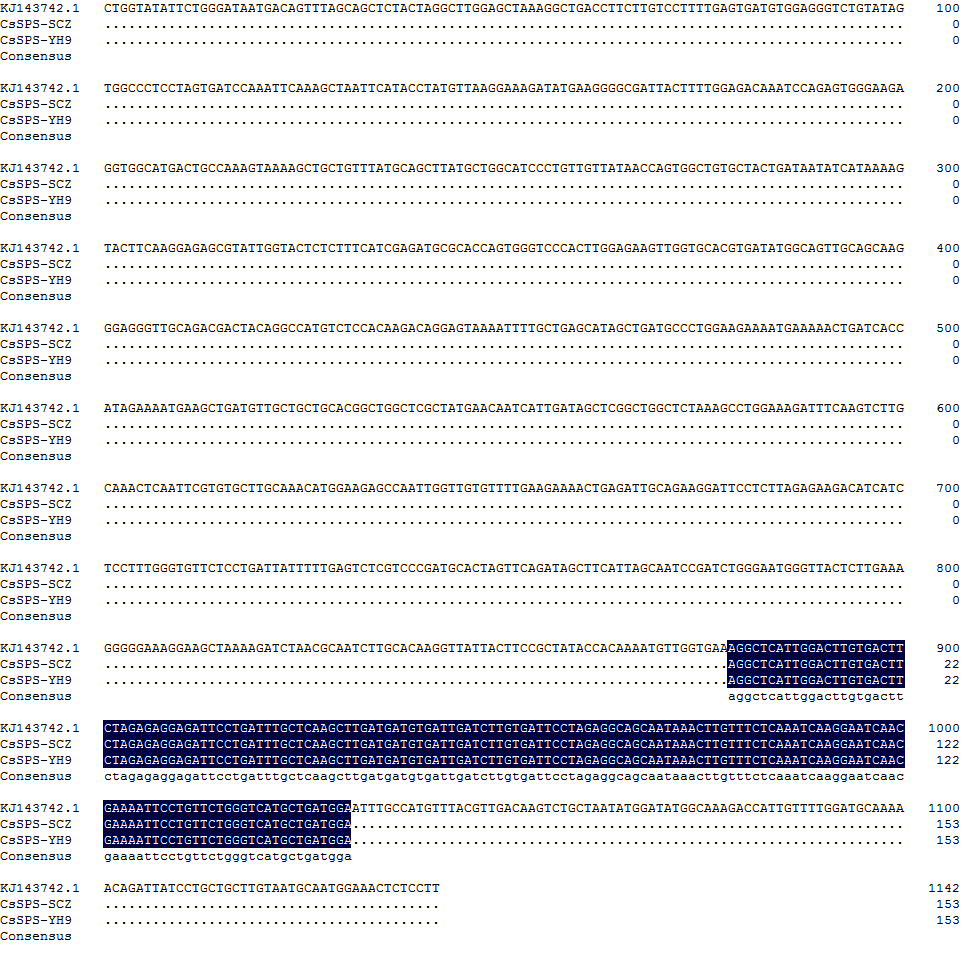
**

**S2. Figure K. Comparative sequence analysis among GenBank Accession and qPCR products of SCZ and YH9 of *CsSPS*.** Identical base sequences are shaded blue.

**
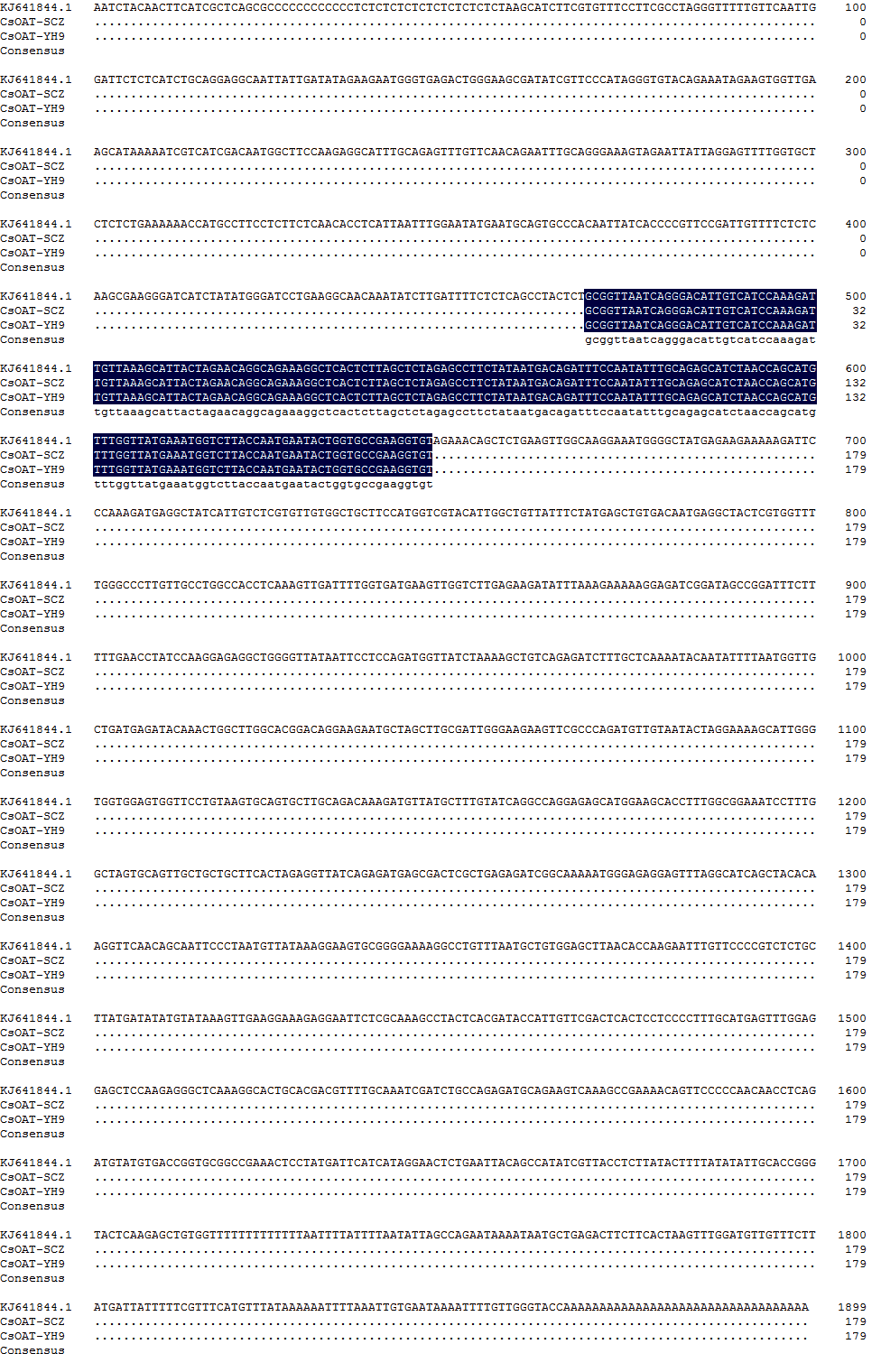
**

**S2. Figure L. Comparative sequence analysis among GenBank Accession and qPCR products of SCZ and YH9 of *CsOAT*.** Identical base sequences are shaded blue.

**
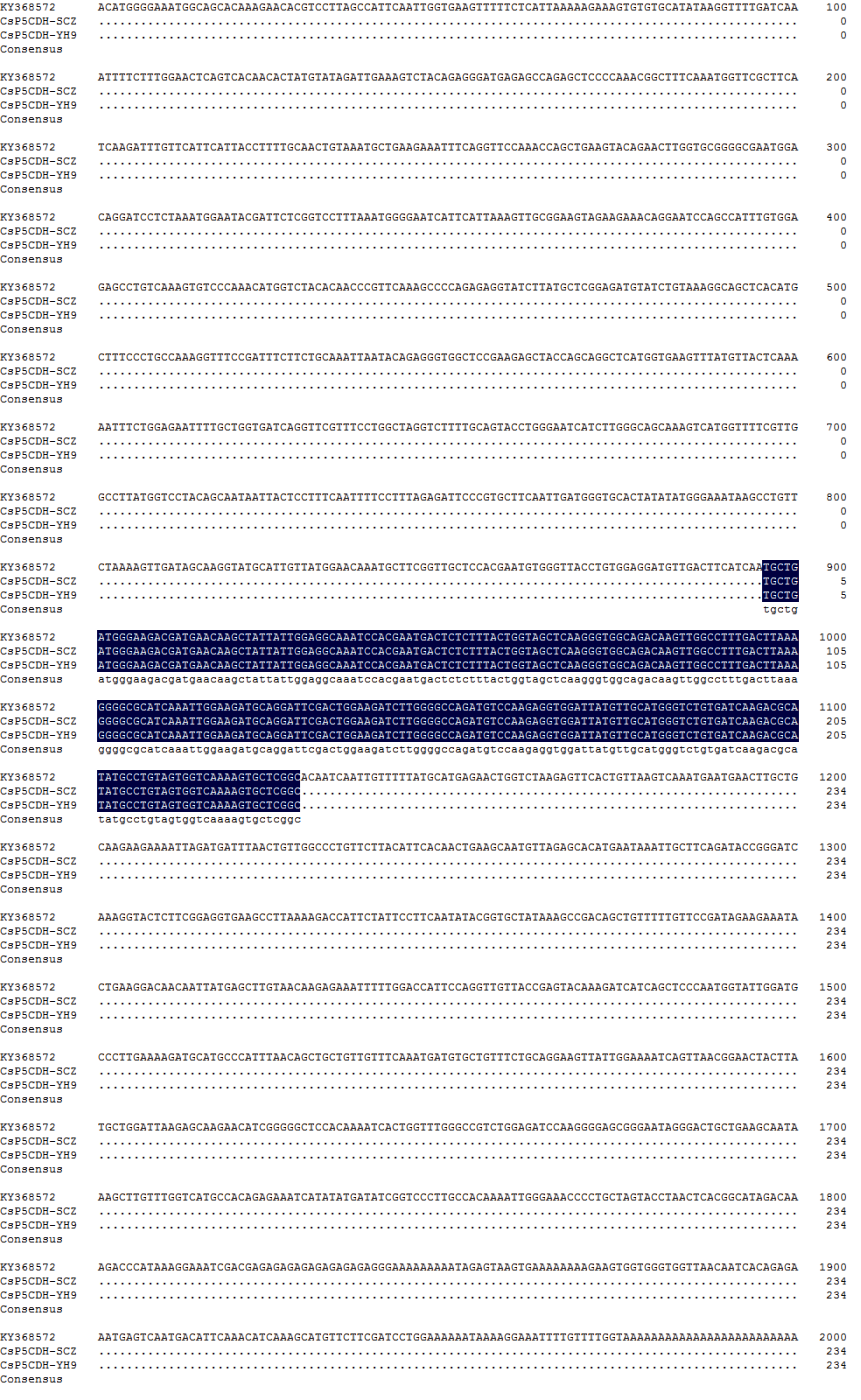
**

**S2. Figure M. Comparative sequence analysis among GenBank Accession and qPCR products of SCZ and YH9 of *CsP5CDH*.** Identical base sequences are shaded blue.

**
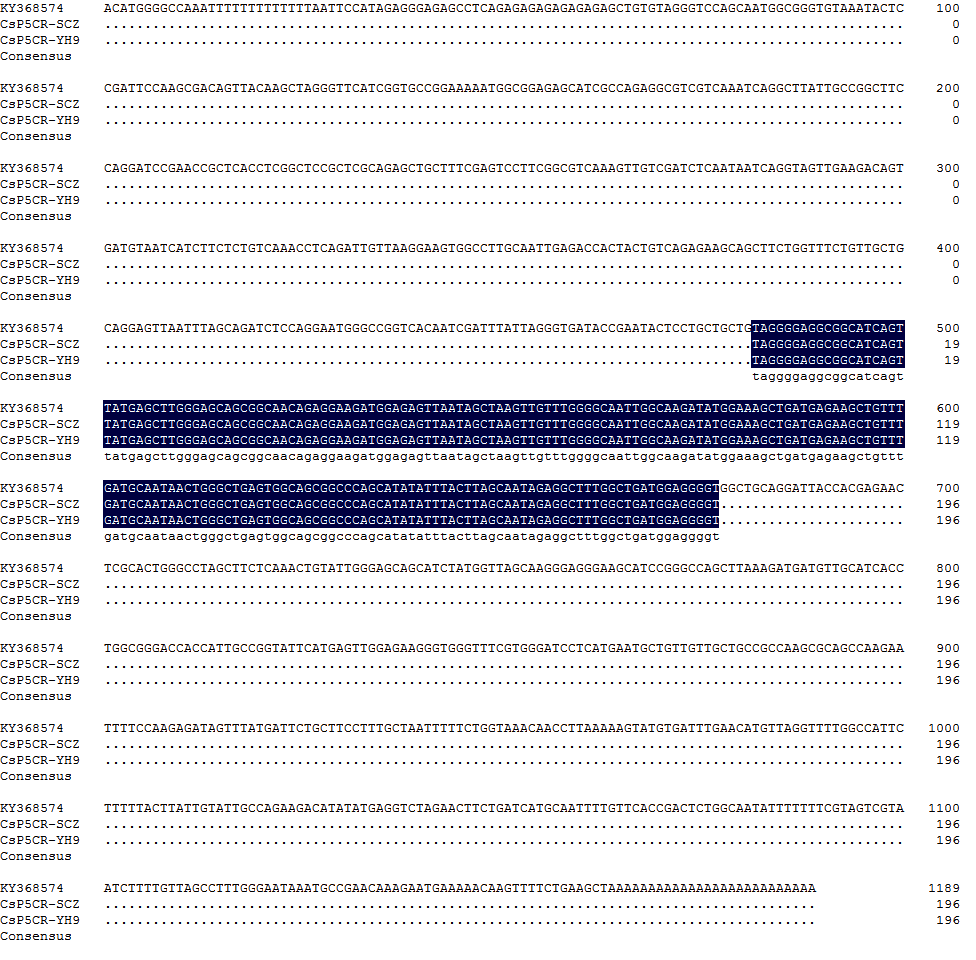
**

**S2. Figure N. Comparative sequence analysis among GenBank Accession and qPCR products of SCZ and YH9 of *CsP5CR*.** Identical base sequences are shaded blue.

**
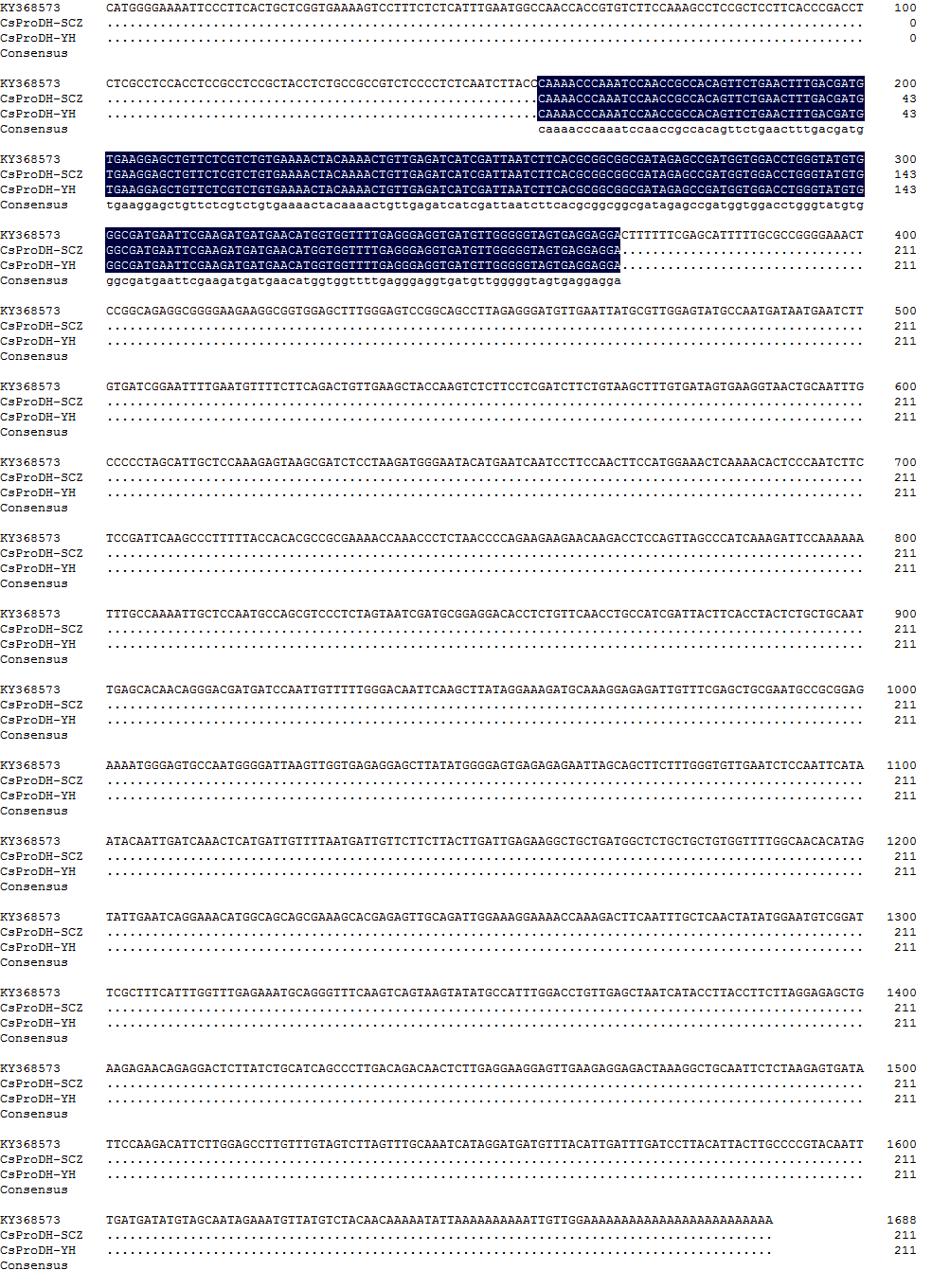
**

**S2. Figure O. Comparative sequence analysis among GenBank Accession and qPCR products of SCZ and YH9 of *CsProDH*.** Identical base sequences are shaded blue.
