# Supplementary material for: Comparative analysis of the response and gene regulation in cold resistant and susceptible tea plants
Source: PLoS One. 2017 Dec 6;12(12):e0188514. doi: 10.1371/journal.pone.0188514 (PMC5718485; doi:10.1371/journal.pone.0188514)
Supplement: S3 Text — (DOCX) [file pone.0188514.s003.docx]

**S3 Text. Effects of CA on freezing tolerance of SCZ**


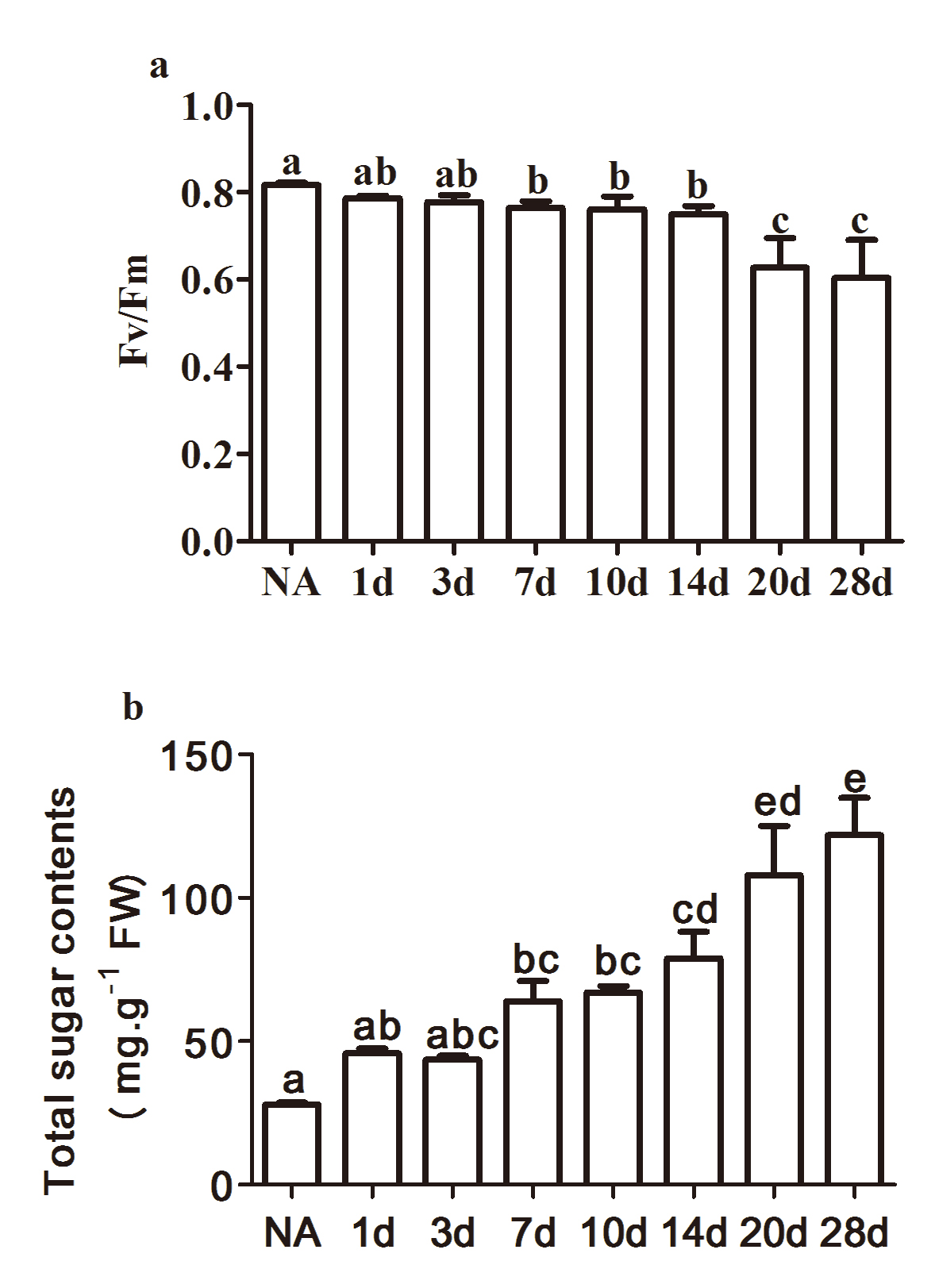


**S3. Figure A. Effects of CA on freezing tolerance of SCZ.** The values Fv/Fm (a) and total sugar contents (b) in SCZ changed in response to cold acclimation of 28 days at 10/4 °C, day/night temperature. Data were displayed as the mean of three replicates with standard error. Columns with different letters in (a) or (b) had significant differences according to Duncan’s multiple range tests with *P* < 0.05. NA: non-acclimation.
